# Noninvasive prediction of axillary lymph node status in breast cancer using promoter profiling of circulating cell-free DNA

**DOI:** 10.1186/s12967-022-03724-w

**Published:** 2022-12-03

**Authors:** Zhi-Wei Guo, Qing Liu, Xu Yang, Geng-Xi Cai, Bo-Wei Han, Li-Min Huang, Chun-Xi Li, Zhi-Kun Liang, Xiang-Ming Zhai, Li Lin, Kun Li, Min Zhang, Tian-Cai Liu, Rui-lin Pan, Ying-Song Wu, Xue-Xi Yang

**Affiliations:** 1 Key Laboratory of Antibody Engineering of Guangdong Higher Education Institutes, School of Laboratory Medicine and Biotechnology, Southern Medical University, 1838 N. Guangzhou Ave, Guangzhou, 510515 China; 2 Department of Breast Surgery, The First People’s Hospital of Foshan, 81 N. North Lingnan Avenue, Foshan, China; 3 Sun Yat-Sen Memorial Hospital, Sun Yat-Sen University, Guangzhou, 510120 China; 4 Department of Respiratory and Critical Care Medicine, Chronic Airways Diseases Laboratory, Nanfang Hospital, Southern Medical University, Guangzhou, 510515 China; 5 Guangzhou XGene Co., Ltd. High-Tech Development Zone, Guangzhou, 510665 People’s Republic of China; 6 Guangzhou First People’s Hospital, The Second Affiliated Hospital of South China University of Technology, Guangzhou, 510180 People’s Republic of China

**Keywords:** Cell-free DNA, Whole-genome sequencing, Promoter profiling, Lymph node metastasis, Breast cancer

## Abstract

**Background:**

Lymph node metastasis (LNM) is one of the most important factors affecting the prognosis of breast cancer. The accurate evaluation of lymph node status is useful to predict the outcomes of patients and guide the choice of cancer treatment. However, there is still lack of a low-cost non-invasive method to assess the status of axillary lymph node (ALN). Gene expression signature has been used to assess lymph node metastasis status of breast cancer. In addition, nucleosome footprint of cell-free DNA (cfDNA) carries gene expression information of its original tissues, so it may be used to evaluate the axillary lymph node status in breast cancer.

**Methods:**

In this study, we found that the cfDNA nucleosome footprints between the ALN-positive patients and ALN-negative patients showed different patterns by implementing whole-genome sequencing (WGS) to detect 15 ALN-positive and 15 ALN-negative patients. In order to further evaluate its potential for assessing ALN status, we developed a classifier with multiple machine learning models by using 330 WGS data of cfDNA from 162 ALN-positive and 168 ALN-negative samples to distinguish these two types of patients.

**Results:**

We found that the promoter profiling between the ALN-positive patients and ALN-negative patients showed distinct patterns. In addition, we observed 1071 genes with differential promoter coverage and their functions were closely related to tumorigenesis. We found that the predictive classifier based on promoter profiling with a support vector machine model, named PPCNM, produced the largest area under the curve of 0.897 (95% confidence interval 0.86–0.93).

**Conclusions:**

These results indicate that promoter profiling can be used to distinguish ALN-positive patients from ALN-negative patients, which may be helpful to guide the choice of cancer treatment.

**Supplementary Information:**

The online version contains supplementary material available at 10.1186/s12967-022-03724-w.

## Background

Lymph node metastasis (LNM) is one of the most important factors affecting the prognosis of breast cancer [1]. The accurate assessment of lymph node status can predict patients’ outcomes and guide the choice of treatment options [2]. Axillary lymph node dissection (ALND) is the gold standard for evaluating axillary lymph node (ALN) status, but it would bring great harm to patients. Although milder sentinel lymph node biopsy (SLNB) has become routine surgery, it is still risk surgery, which would increase considerable anesthesia time and expense, and cause multiple complications in 3.5–10.9% of patients [3, 4]. Therefore, developing a low-cost non-invasive method to evaluate the status of ALN would be of great benefit to breast cancer patients.

Cell-free DNA (cfDNA) has been an essential biomarker in many cancer applications, such as early detection and outcome prediction of cancer [5]. At present, the most commonly used features are cfDNA level and its sequence information. Previous studies have described the close relationship between abnormal cfDNA levels and ALN metastasis [6, 7], which indicates that cfDNA may be used to assess ALN status. However, the level of cfDNA is influenced levels are affected by many pathological processes, such as infection and inflammation [8–10]. In addition, some studies wanted to find ALN metastasis-related ctDNA mutations or ctDNA hypermethylation [1, 11–13], however, no relationship was found between them [2]. Thus, novel disease-specific features of cfDNA with high predictive efficacy are needed to be found for predicting LNM.

Recently, cfDNA coverage on gene promoter has found that it carried gene expression information of its original tissues [14, 15]. Plasma cfDNA is mainly released by apoptotic cells after enzymatic processing of chromatin [16]. The DNA bound to the nucleosomes is retained, while the exposed DNA between the nucleosomes is digested. Analysis of cfDNA fragments derived from cancers showed that the promoter regions of active genes exhibited depleted coverage, which meant that nucleosome binding was less in these regions along with increased gene expression [15]. In cancer patients, cfDNA is mainly derived from tumor and hematopoietic cells [16]. More importantly, studies on breast cancer have shown that many gene expression signatures could be used to estimate the risk of distant relapses, and some of which have been commercialized, such as PAM50. In addition, the immune cells have been proved to play an important role in tumor metastasis, and the peripheral blood immunome of breast cancer patients is influenced by the existence and stage of cancer [17, 18]. Therefore, we assume that the cfDNA coverage at the gene promoter has potential to assess the ALN status.

In this study, we first compared the nucleosome footprint around the transcriptional start sites (TSS) of ALN-positive and ALN-negative breast cancer patients to identify genes with differential coverage. In order to further evaluate the potential of promoter profiling for evaluating ALN status, we developed a classifier for distinguishing ALN-positive and ALN-negative patients by using multiple machine learning models. Finally, we incorporated some clinicopathological characteristics in our classifier to test whether its performance would improve.

## Methods

### Participants and study design

From January 2018 to December 2019, before cancer therapy, plasma samples were prospectively collected from 330 breast cancer patients, including 162 ALN-positive and 168 ALN-negative patients. We excluded patients who: (1) were pregnant or lactating, (2) were metastatic breast cancer or had non-infiltrating tumors histologically, (3) had a hematopoietic system or inflammatory breast diseases, and (4) were ALN-negative patients diagnosed with fine needle aspiration biopsy. We reviewed all tumor specimens histopathologically and staged them according to the seventh edition of the American Joint Committee on Cancer (AJCC) staging system for breast cancer. All plasma samples were obtained under institutional review board of The First People's Hospital of Foshan approved protocols with written informed consent from all participants for research use (ID: L[2021]-7). Table 1 summarizes the characteristics of patients, including age, T stage, estrogen- (ER) and progesterone-receptor (PR) status, expression of human epidermal growth factor receptor 2 (Her2), proliferative fraction (Ki-67 labeling index), and histological grade.

**Table 1:** Patient characteristics

	ALN-positive (n = 162)			ALN-negative (n = 168)		
	Training (n = 113)	Validation (n = 49)	*P*-value	Training (n = 118)	Validation (n = 50)	*P*-value
Age						
Years [range]	51.6 [31–83]	49.1 [35–83]	0.124^a^	51.7 [28–88]	52.9 [26–79]	0.611^a^
T stage						
T1	22 (19.5)	13 (26.5)	0.355^b^	77 (65.3)	28 (56.0)	0.288^c^
T2	69 (61.1)	24 (49.0)		39 (33.1)	22 (44.0)	
T3/T4	22 (19.4)	12 (24.5)		2 (1.6)	0 (0)	
ER						
Positive	90 (79.6)	43 (87.8)	0.311^b^	99 (83.9)	40 (80.0)	0.698^b^
Negative	23 (20.4)	6 (12.2)		19 (16.1)	10 (20.0)	
PR						
Positive	94 (83.2)	39 (79.6)	0.745^b^	98 (83.1)	41 (82.0)	1^b^
Negative	19 (16.8)	10 (20.4)		20 (16.9)	9 (18.0)	
Her2						
Positive	97 (85.8)	38 (77.6)	0.284^b^	97 (82.2)	42 (84.0)	0.953^b^
Negative	16 (14.2)	11 (22.4)		21 (17.8)	8 (16.0)	
Ki67						
< 20	41 (36.3)	21 (42.9)	0.539^b^	63 (53.4)	21 (42.0)	0.238^b^
≥ 20	72 (63.7)	28 (57.1)		55 (46.6)	29 (58.0)	
Histological grade						
1	3 (2.7)	4 (8.2)	0.174^c^	14 (11.9)	4 (8.0)	0.640^c^
2	82 (72.6)	37 (75.5)		80 (67.8)	38 (76.0)	
3	28 (24.7)	8 (16.3)		24 (20.3)	8 (16.0)	

Data is showed as patient number (%)

*ER* estrogen receptor, *PR* progesterone receptor, *Her2* human epidermal growth factor receptor 2

^a^Wilcox rank sum test

^b^Chi square test

^c^Fisher’s exact test

### ALN surgery

The ALN status was ascertained clinically by fine needle aspiration biopsy, ALND or SLNB. Because the number of lymph nodes detected by the fine needle aspiration biopsy is limited, some positive lymph nodes may be ignored, which may increase the false positive rate of the evaluation model. Therefore, the patients with ALN-negative detected by fine needle aspiration biopsy were excluded from this study. Indocyanine green with a carbon nanoparticle suspension was used for SLNB and more than three LNs were checked for cancer.

### Extracting and sequencing cfDNA

In total, 1 mL peripheral blood was collected using EDTA tubes from each patient and then immediately implemented two-step centrifugation to obtain the plasma. The centrifugation parameters were is 1600*g* for 10 min, followed by 10 min at 16,000*g* at 4 °C. Subsequently, the plasma was stored at − 80 °C before use. Each sample yielded at least 1 ng total cfDNA for sequencing. cfDNA was extracted from plasma by QIAamp DNA Blood Mini Kit (Qiagen). A starting amount of approximately 1–5 ng DNA was used for library construction with the Life Sciences Ion Xpress™ Plus Fragment Library Kit. The number of PCR cycles was set to 12. The DNA size distribution of libraries was analyzed on a Bioanalyzer instrument (Agilent Technologies, Singapore). Sequencing was performed with the Ion PI™ Hi-Q™ OT2 200 Kit and the Ion PI™ Hi-Q™ Sequencing 200 Kit on Ion Proton platform (ThermoFisher Scientific, USA) with 520 flow. The mean depth of the sequencing samples was approximately 0.3×.

### Sequencing data processing

After sequencing, the raw read was aligned to the human reference genome (hg19) using bwa (ver.0.7.5). Then, SAMtools rmdup function (ver. 0.1.18) was used to remove the polymerase chain reaction duplicates [19]. The GC-bias correction was implemented using the deeptools (ver.3.5.0) with the default setting. The calculation of tumor fraction and copy number-bias correlation were implemented using ichorCNA algorithm [20].

### Promoter profiling calculation

The calculation of promoter profiling was similar to that used in our previous study [15, 21]. In briefly, gene information was downloaded from RefSeq of University of California Santa Cruz [22]. The region ranging from − 1 KB to + 1 KB around the transcriptional start site of each transcript, was defined as the primary transcription start site (pTSS), was first identified. The read counts for each base at the pTSS were calculated using DANPOS with default setting [23]. After read alignment, the read coverage at the pTSS was extracted from the aligned BAM files using bedtools (ver. 2.17.0). Then, the read coverage was normalized by the reads per kilobase per million mapped reads (RPKM)-like method. The normalized value of promoter profiling was calculated by the following formula:

$$Normailzed \,Promoter \, profiling=\frac{\mathrm{cfDNA \, coverage \, around \, TSS}\times \mathrm{ 1,000,000}}{\mathrm{Totally \,mapped \,reads }\times \mathrm{ length}},$$

here, the length of each transcript is equal to 2000 because of the pTSS region ranging from − 1 KB to + 1 KB around each transcriptional start site.

### Models for evaluating lymph node status

To develop the evaluation classifier, the patients were firstly divided into three cohorts, including discovery, training and validation cohorts. In the discovery cohort, we identified the genes with differential promoter coverage. Then, the plasma samples were then divided into training and validation cohorts in a ratio of 7:3. Based on the training cohort data, we developed classifiers using three models, including support vector machine (SVM), logistic regression (LR), and linear discriminant analysis (LDA) models, to distinguish ALN-positive and ALN-negative tumors. The importance of the features was assessed with the sigFeature package of R. Then we selected top 100 features for further classifier construction. The SVM classifier was constructed with the linear kernel in e1071 package using the default setting. In order to identify the optimal gene combination with the largest area under the curve (AUC), backward method was adopted. To avoid potential bias and over-fitting in the training cohort, the leave-one-out cross validation method was used to evaluate the robustness of these classifiers. Briefly, each subject in the training cohort was withheld in turn, and the rest subjects were submitted to train the model. The trained model was then used to determine the class of the withheld subject. This procedure went on until all subjects in the training cohort were judged. Finally, the efficacy of selected classifiers was evaluated using the validation cohort data.

### Statistical analysis

Wilcoxon rank-sum test or Chi square test were used for analyses that compared the two groups. Benjamini–Hochberg method was used to adjust the raw *P*-values to the false discovery rate (FDR). Variables with fold change ≥ 1.5 and FDR ≤ 0.05 were considered statistically significant. The genes with differential promoter coverage were used to plot uniform manifold approximation and projection (UMAP) and heat map using uwot package and pheatmap package in R (version 3.0.1), respectively. Receiver operating characteristic (ROC) curves were plotted and differences in the AUC were compared using the pROC package [24]. GO enrichment analysis was implemented by using Metascape with default settings [25]. Housekeeping genes and non-constitutive genes were downloaded from the additional material of a previous study [14].

## Results

### cfDNA promoter profiling related to tumor expression profiles

**Fig. 1:**
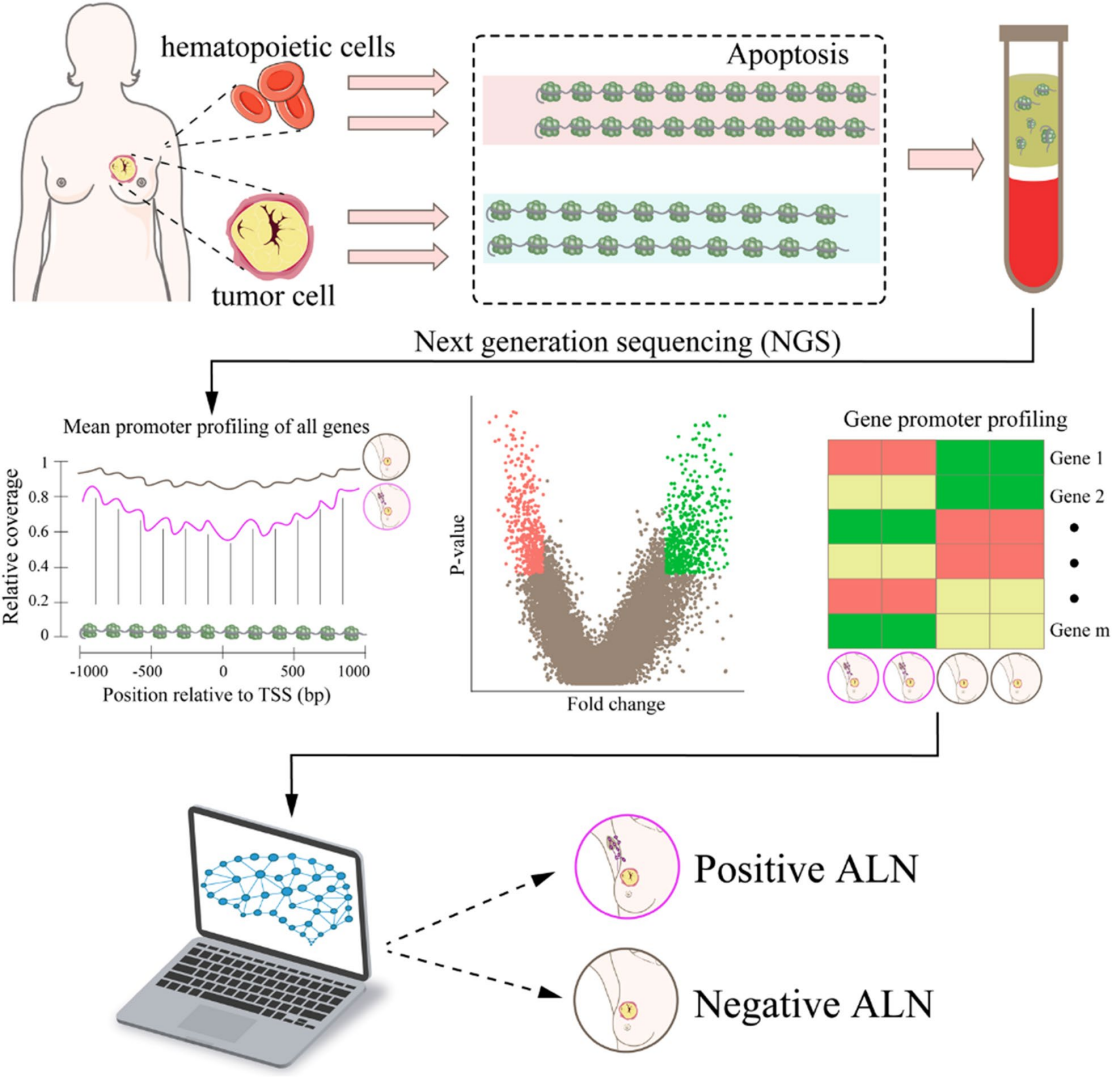
Schematic diagram of PPCNM. In cancer, plasma cell-free DNA (cfDNA) is primarily derived from apoptotic tumor and hematopoietic cells. Exposed DNA not bound to a nucleosome is digested, whereas nucleosome-bound DNA escapes digestion and enters the circulation. cfDNA has a nucleosome footprint, which carries information about its original tissues and could reflect its gene expression status. Because axillary lymph node (ALN)-positive and ALN-negative breast cancer patients have different gene expression signatures in tumor and hematopoietic cells, their nucleosome patterns may show difference. Therefore, we assume that the promoter coverage of cfDNA detected by whole-genome sequencing could be used to develop classifiers for predicting lymph node metastasis

In order to test whether the promoter profiling of cfDNA could be used to predict ALN metastasis, we first studied whether the coverage of gene promoter regions (± 1 KB around TSS) was related to gene expression profiles (Fig. 1). Consistent with previous studies [14], the promoter coverage of housekeeping genes with high expression levels was significantly reduced compared with those of non-constitutive genes (Fig. 3a). Then, we studied whether the footprint of nucleosomes around the TSS was different between ALN-positive and ALN-negative groups. In ALN-positive breast cancer patients, we observed the loss of related cfDNA signals (Fig. 3b; *P* = 2.2e−16, Wilcoxon rank sum test).

### Genes with differential promoter coverage associated with LNM

**Fig. 2:**
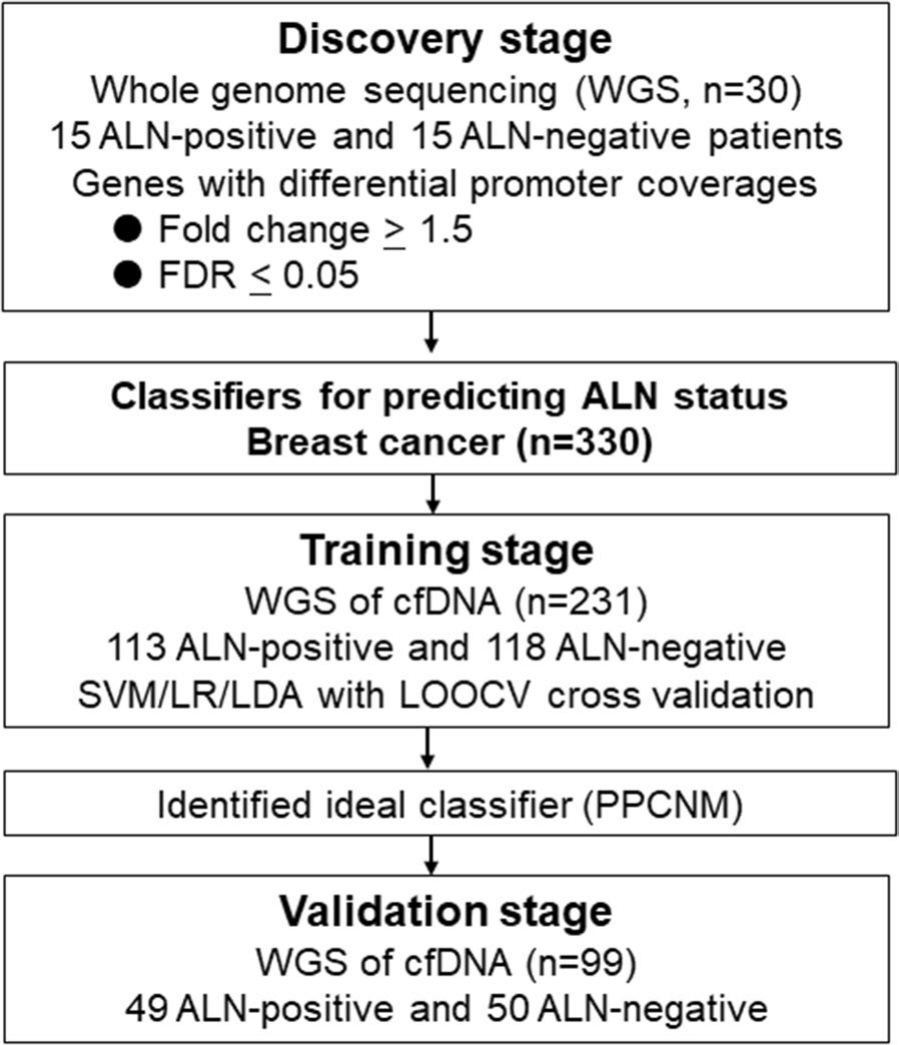
Study design. In order to develop classifiers to predict ALN status, our study was divided into three stage, including discovery, training and validation stage. In the discovery stage, the genes with differential coverages were identified. In the training stage, different machine learning models were used to develop classifiers by using the differential features. The importance of the features was assessed with the sigFeature package of R. Then we selected top 100 features for further classifier construction. In order to identify the optimal gene combination with the largest area under the curve (AUC), backward method was adopted. Finally, the classifiers with the largest AUC were selected. In the validation stage, the predictive efficacy of the selected classifiers was assessed using an internal validation cohort. The detailed characteristics of breast cancer patients were shown in Table 1. *WGS* whole genome sequencing, *ALN* axillary lymph node, *TSS* transcriptional start site, *SVM* support vector machine, *LR* logistic regression, *LDA* linear discriminant analysis, *LOOCV* leave one out cross validation

**Fig. 3:**
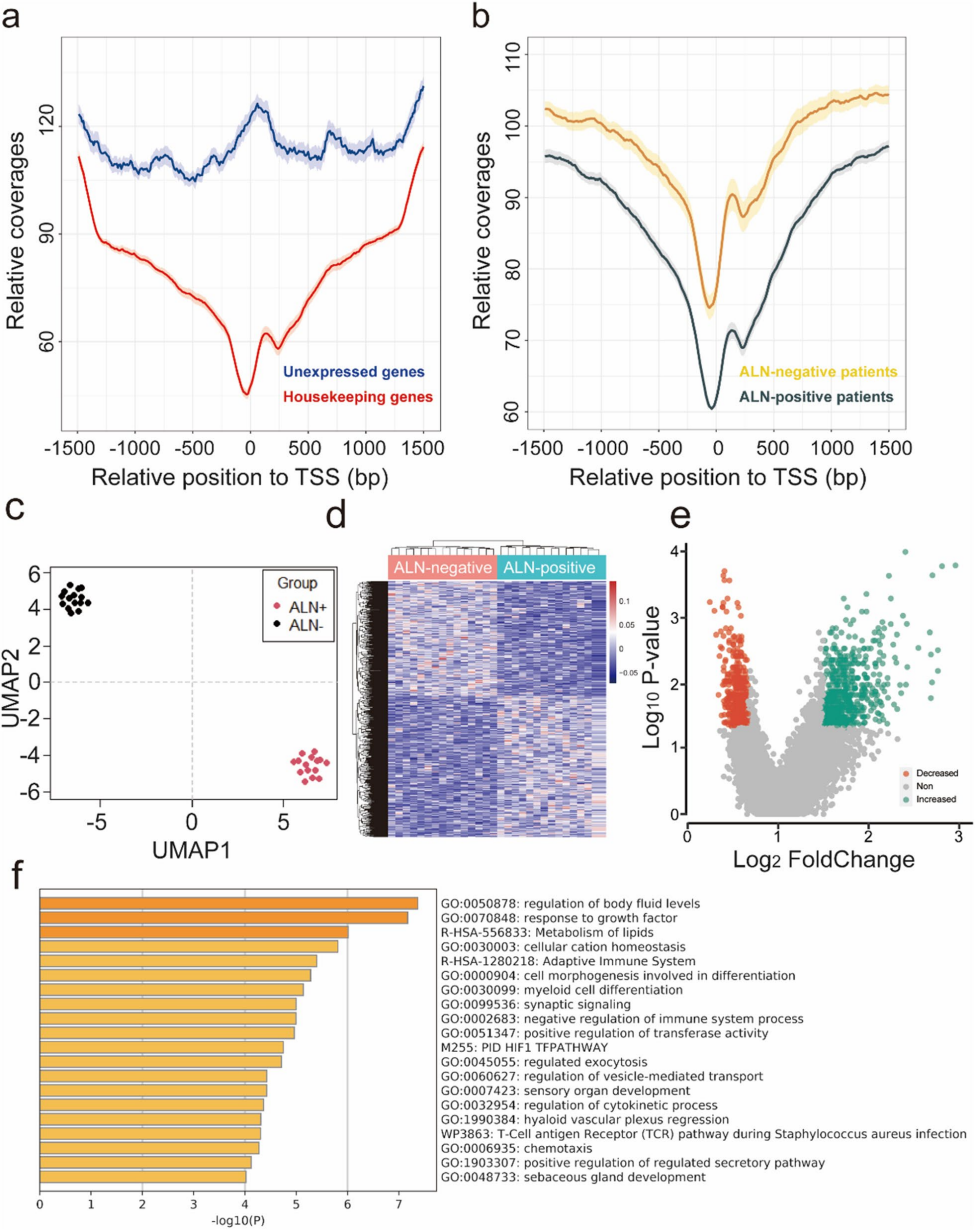
The cfDNA promoter profiling shows the potential to predict ALN status. **a** Promoter profiling of the non-constitutive and housekeeping genes. The average promoter coverage was calculated by using the whole genome sequencing data derived from 30 breast cancer patients. The non-constitutive and housekeeping genes were obtained from the additional materials of previous study [14]. **b** Promoter profiling of the ALN-negative and -positive breast cancer patients. Mean promoter profiling of protein coding genes derived from15 ALN-positive and 15 ALN-negative breast cancer patients was detected using whole genome sequencing. **c** Uniform manifold approximation and projection (UMAP) plot representing the associations between ALN-positive and -negative groups. The genes with differential promoter coverages were used to plot UMAP. **d** Heat map of the z-scores of genes with differential read coverages. **e** Volcano plots of gene transcripts with differential read coverages at the promoter (fold change ≥ 1.5 and false discovery rate [FDR] ≤ 0.05) between 15 ALN-positive and 15 ALN-negative patients. **f** Analysis of Gene Ontology (GO) enrichment of genes with differential promoter coverage. *TSS* transcriptional start sites, *Decreased* genes with differentially decreased read coverage, *Increased* genes with differentially increased read coverage, Non genes with no differential read coverage, *ALN* axillary lymph node

The workflow of our study mainly consisted of three stages, including discovery, training and validation stages (Fig. 2). In the discovery cohort, we identified the genes with differential promoter coverage. When comparing the promoter profiling of each gene, we observed 1,071 genes with differential promoter coverage between ALN-positive and ALN-negative patients (Fig. 3e and Additional file 1: Table S1; fold change ≥ 1.5 and FDR ≤ 0.05, Wilcoxon rank sum test). Then, using UMAP, we found that samples from the same groups were clustered together, while the samples from different groups were scattered (Fig. 3c). In addition, the heat map showed distinct patterns of promoter coverage between ALN-positive and ALN-negative breast cancer patients (Fig. 3d). These results indicated that promoter profiling has potential for assessing the ALN status of breast cancer.

By GO enrichment analysis of the genes with differential promoter coverage, we found that most of GO terms were immuno-associated and growth-associated processes (Fig. 3f). Consistent with the existing literature, cfDNA could reflect the expression status of its original tissues. As the expression of tumor and peripheral blood immunome was closely related to cancer stage [18], the above annotation results may indicate that the genes with differential promoter coverage may be associated with ALN involvement.

### Classifiers for evaluating ALN status

To evaluate the potential of promoter profiling for assessing ALN status, we used WGS to characterize the promoter profiling of cfDNA derived from 330 breast cancer patients collected from January 2018 to December 2019, including 162 ALN-positive and 168 ALN-negative patients. The patients were split into training and validation cohorts with a 7:3 ratio and the clinicopathological parameters, such as age, T stage, ER, PR, and Her2 status, were well balanced between the two cohorts of breast patients (Table 1; all *P* > 0.05).

**Fig. 4:**
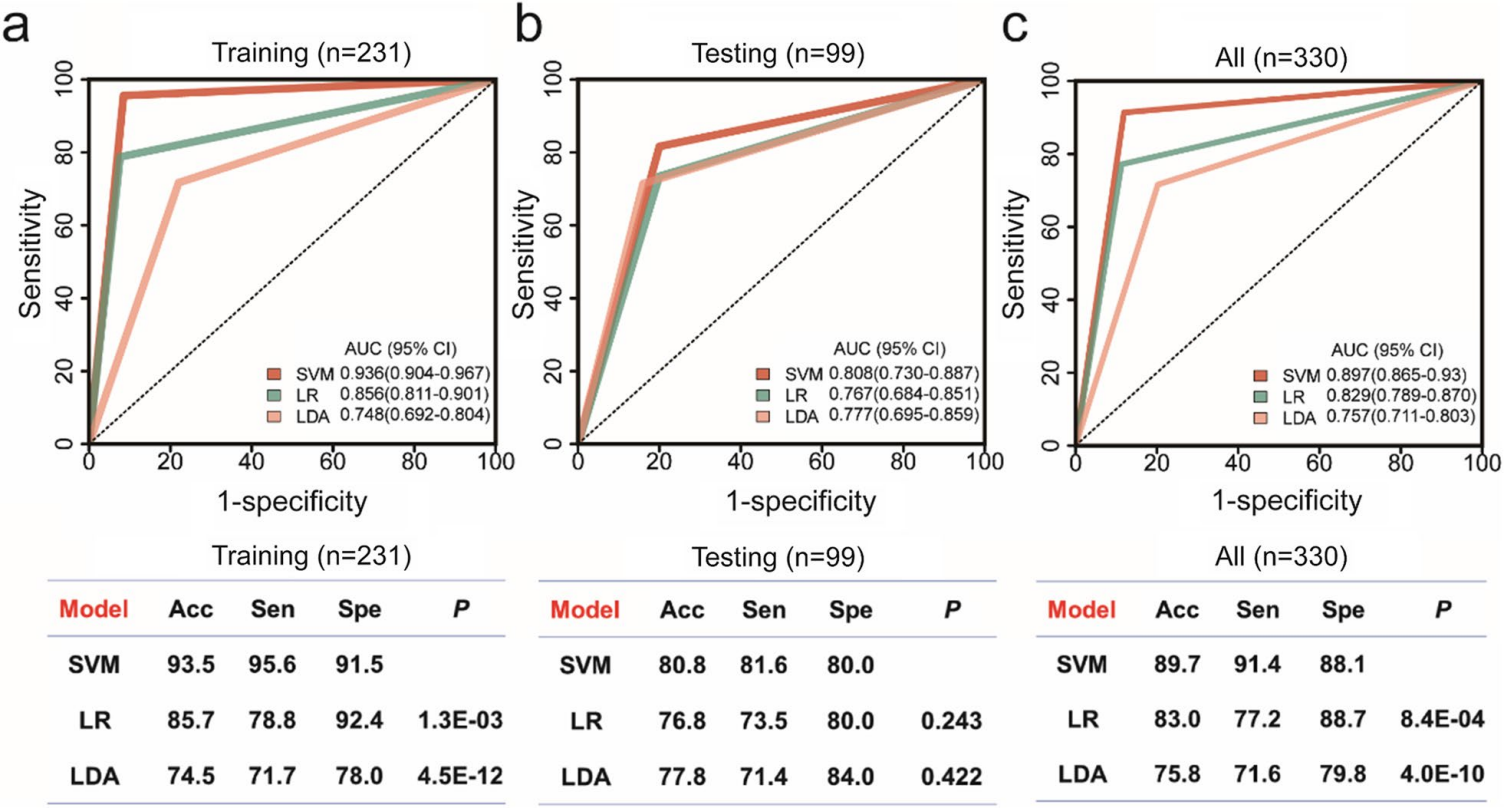
Receiver operating characteristic (ROC) curves of PPCNM**. a,** Support vector machine, SVM **b**, Logistic regression, LR. **c**, Linear discrimination analysis, LDA. *Acc* accuracy, *Sen* sensitivity, *Spe* specificity, *P* the *P* value of AUC comparison between SVM vs. LR and SVM vs. LDA calculated by pROC package in R. The ROC showed the AUC of the best combination after cross-validation, therefore, it lacked the ‘arc’ shape

Then, we used genes with differential promoter coverage in SVM model to develop classifiers to distinguish ALN-positive from ALN-negative patients. ROC analysis was used to evaluate the AUC, sensitivity, specificity and accuracy of the promoter profiling classifiers (Fig. 4a). Among these combinations, a 48-gene combination named PPCNM performed well in the training cohort after LOOCV, with an AUC of 0.936 (95% confidence interval [CI] 0.904–0.967 and an accuracy of 0.848, Fig. 4a and Additional file 1: Table S2). The performance of PPCNM was further evaluated in the validation cohort, and we found that the AUC of PPCNM in the validation cohort was 0.808 (0.730–0.887) (Fig. 4b). These results indicated that a classifier based on promoter profiling can be used to assess ALN status.

Across all cohorts, the average AUC of PPCNM was 0.897 (0.865–0.930), which was used to distinguish ALN-positive and ALN-negative patients, with a sensitivity of 0.914 and a specificity of 0.881 (Fig. 4c). The AUC produced by PPCNM was significantly greater than those of classifiers based on the LR and LDA models (Fig. 4c, LR: 0.829 [0.789–0.870], *P* = 8.37E−04 and LDA: 0.757 [0.711–0.803], *P* = 4.03E−10).

### PPCNM and tumor DNA fraction

The level of tumor DNA fraction is one of the most important characteristics of tumor. Firstly, we calculated the tumor DNA fraction of ALN-positive and ALN-negative patients, and found that its levels between these two groups were similar (Additional file 1: Fig. S1; *P*-value = 0.1663). In addition, we found that the efficacy of PPCNM was similar in different concentrations of tumor DNA fraction (all *P*-value > 0.5; Additional file 1: Table S3). The AUC of tumor DNA fraction used to predict ALN status was 0.544 (0.482–0.606). The efficacy of the combination of PPCNM with tumor DNA fraction has an AUC 0.845 (0.806–0.885), which is significantly lower than that of PPCNM (*P* = 2.8E−04).

### PPCNM combined with clinicopathological characteristics

**Fig. 5:**
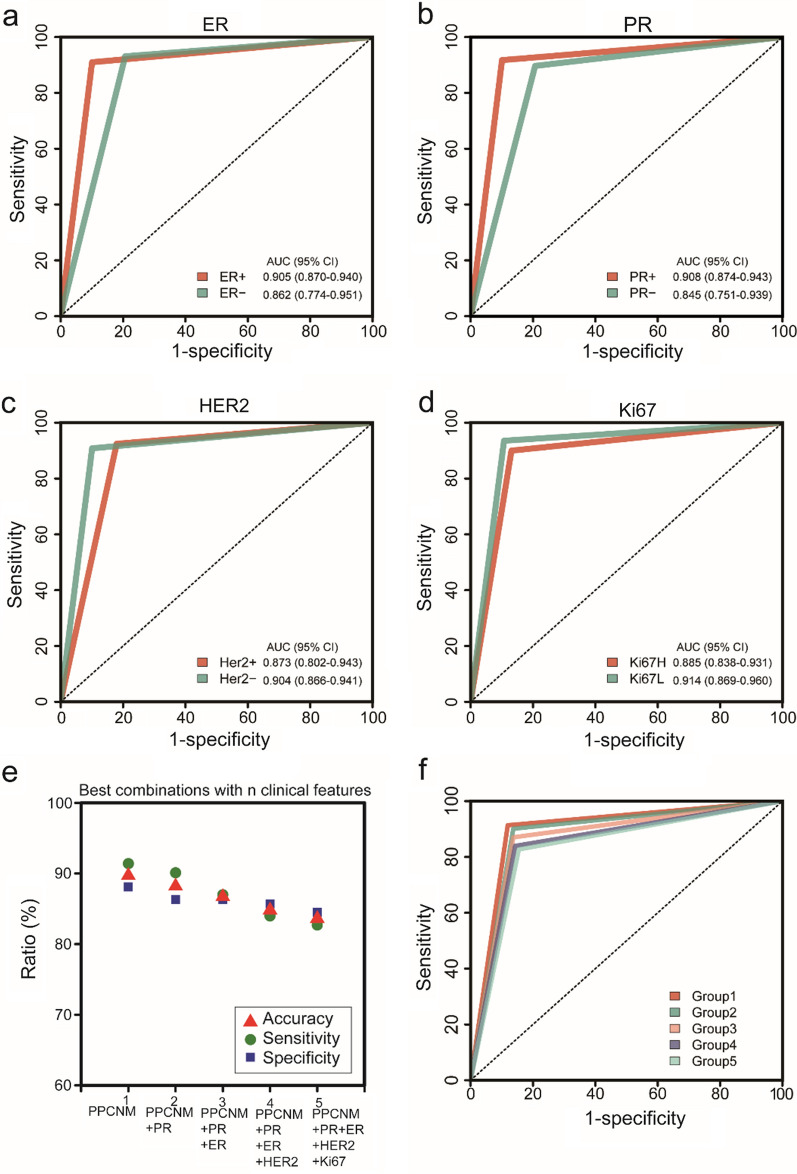
Performance of classifiers. **a** ROC curve for (ER/PR/Her2/Ki67)-positive and (ER/PR/Her2/Ki67)-negative groups. **b** Performance of the best combinations of PPCNM with different number of clinical features. **c** ROC curve for the best combinations of PPCNM with different number of clinical features. *AUC* area under curve, *SVM* support vector machine, *LR* logistic regression

Previous studies have shown the close relationship between ER, PR, Her2, and Ki67 status with ALN metastasis [26, 27]. Therefore, we first investigated whether the efficacy of our classifiers was different between positive- and negative-status of each feature. The efficacy of the PPCNM model was similar in regards to ER-positive vs. ER-negative, PR-positive vs. PR-negative, Her2-positive vs. Her2-negative, and Ki67-High and Ki67-Low (Fig. 5a–d). We then incorporated these clinical characteristics with PPCNM to see whether its performance would further improve. By evaluating the efficacy of their exhaustive combination with PPCNM, we found that the AUC, accuracy, sensitivity of the PPCNM decreased after being combined with one of the four clinical features (Fig. 5e, f and Additional file 1: Table S4).

## Discussion

We found that there was a significant difference in promoter profiling between ALN-positive and ALN-negative breast cancer patients (Fig. 3). The classifier PPCNM based on promoter profiling using the SVM model, produced the maximum AUC (0.897 [0.865–0.930]) for distinguishing these two groups of patients, and its performance was significantly better than those of classifiers relied on LR and LDA regression models (Fig. 4c; all *P* < 0.05). In addition, the AUC increased slightly with the incorporation of clinical characteristics. These findings indicate that PPCNM may be a promising non-invasive tool for evaluating ALN status.

There are forty-eight genes in the PPCNM (Additional file 1: Table S2). These genes are closely associated with the metastasis of tumor. For instance, a large number of studies have reported the close relationship between NF-κB signaling pathway and tumor metastasis [28, 29]. NF-κB signaling pathway regulates the expression of its downstream target genes, including MMP9, TNFα, uPA and IL8, thus promoting the invasion and metastasis of breast cancer cells [29]. Besides, BHLHE40 confers a pro-survival and pro-metastatic phenotype to breast cancer cells by modulating HBEGF secretion [30]. And BHLHE40 facilitates the invasion of cancer cell by interacting with SP1 [31]. In addition, USP20 can promote breast cancer metastasis by stabilizing SNAI2 [32].

ALN status is an essential factor for the prognosis of breast cancer patients and the choice of cancer treatment in breast cancer [2]. Although milder SLNB has become more pervasive, LN surgery for evaluating ALN status still brings various side effects to patients. Therefore, developing a non-invasive method to predict ALN status may be beneficial to breast cancer patients. At present, some studies show that increased cfDNA levels are related to ALN Metastasis [6, 7]. But cfDNA levels were affected by various physiological and pathological processes [8–10]. More specific features of cfDNA have to be found for assessing ALN status. Previous studies have reported that cell-free DNA promoter profiling and TF profiling is capable of prediction of tumor subtypes in prostate and detect early-stage colorectal cancer [14, 33]. Therefore, we assume that promoter profiling could be used to evaluate ALN status. In this study, we found the characteristics of specific promoter profile signatures of cfDNA in ALN-positive and ALN-negative patients (Fig. 3e). The classifiers (PPCNM) based on these differential variables achieved high performance with an AUC of 0.897 [0.865–0.930]. We developed a non-invasive method based on plasma cfDNA to assess ALN status, which could dynamically monitor the status of lymph node. More importantly, our method could avoid the heterogeneity of tumor in tissue detection. Nevertheless, there are some limitations in our research. Although the AUC of our classifier achieved 0.897, and 330 WGS data was used in this study, more prospective samples and samples from other external centers were needed to improve the predictive value of efficacy before clinical application.

In summary, our data suggest that PPCNM is a promising tool based on promoter profiling for evaluating ALN status in breast cancer. PPCNM is a non-invasive technique, which only needs low-coverage DNA sequencing and is not affected by cancer heterogeneity. Therefore, the PPCNM classifier may help patients and clinicians to choose appropriate cancer treatment methods, thus improving the curative effects and the quality of life of cancer.

## Supplementary Information

**Additional file 1: Figure S1.** The tumor DNA fraction between ALN-positive and ALN-negative patients. **Table S1.** Genes with differential promoter coverage between ALN-positive and -negative groups. **Table S2.** The genes in PPCNM. **Table S3.** The predictive efficacy of PPCNM in different tumor fraction. **Table S4.** Predictive efficacy of the PPCNM with characteristics.

## Data Availability

All datasets generated for this study are included in the article/additional material.

## Abbreviations

LNM: Lymph node metastasis
ALND: Axillary lymph node dissection
SLNB: Sentinel lymph node biopsy
cfDNA: Cell-free DNA
WGS: Whole genome sequencing
TSS: Transcriptional start site
ER: Estrogen receptor
PR: Progesterone receptor
HER2: Human epidermal growth factor receptor 2
pTSS: Primary transcription start site
SVM: Support vector machine
LR: Logistic regression
LDA: Linear discriminant analysis
LOOCV: Leave one out cross-validation
AUC: Area under curve
UMAP: Uniform manifold approximation and projection

## References

[1] Fiegl H, Millinger S, Mueller-Holzner E, Marth C, Ensinger C, Berger A, Klocker H, Goebel G, Widschwendter M (2005). Circulating tumor-specific DNA: a marker for monitoring efficacy of adjuvant therapy in cancer patients. Cancer Res.

[2] Lee JH, Jeong H, Choi JW, Oh HE, Kim YS (2018). Liquid biopsy prediction of axillary lymph node metastasis, cancer recurrence, and patient survival in breast cancer: a meta-analysis. Medicine.

[3] Boughey JC, Moriarty JP, Degnim AC, Gregg MS, Egginton JS, Long KH (2010). Cost modeling of preoperative axillary ultrasound and fine-needle aspiration to guide surgery for invasive breast cancer. Ann Surg Oncol.

[4] Langer I, Guller U, Berclaz G, Koechli OR, Schaer G, Fehr MK, Hess T, Oertli D, Bronz L, Schnarwyler B (2007). Morbidity of sentinel lymph node biopsy (SLN) alone versus SLN and completion axillary lymph node dissection after breast cancer surgery: a prospective Swiss multicenter study on 659 patients. Ann Surg.

[5] Cristiano S, Leal A, Phallen J, Fiksel J, Adleff V, Bruhm DC, Jensen SO, Medina JE, Hruban C, White JR (2019). Genome-wide cell-free DNA fragmentation in patients with cancer. Nature.

[6] Agassi R, Czeiger D, Shaked G, Avriel A, Sheynin J, Lavrenkov K, Ariad S, Douvdevani A (2015). Measurement of circulating cell-free DNA levels by a simple fluorescent test in patients with breast cancer. Am J Clin Pathol.

[7] Payne RE, Hava NL, Page K, Blighe K, Ward B, Slade M, Brown J, Guttery DS, Zaidi SA, Stebbing J (2012). The presence of disseminated tumour cells in the bone marrow is inversely related to circulating free DNA in plasma in breast cancer dormancy. Br J Cancer.

[8] Yi J, Zhang Y, Ma Y, Zhang C, Li Q, Liu B, Liu Z, Liu J, Zhang X, Zhuang R, Jin B (2014). Increased plasma cell-free DNA level during HTNV infection: correlation with disease severity and virus load. Viruses.

[9] Tovbin D, Novack V, Wiessman MP, Abd Elkadir A, Zlotnik M, Douvdevani A (2012). Circulating cell-free DNA in hemodialysis patients predicts mortality. Nephrol Dial Transplant.

[10] Tsai NW, Lin TK, Chen SD, Chang WN, Wang HC, Yang TM, Lin YJ, Jan CR, Huang CR, Liou CW, Lu CH (2011). The value of serial plasma nuclear and mitochondrial DNA levels in patients with acute ischemic stroke. Clin Chim Acta.

[11] Beaver JA, Jelovac D, Balukrishna S, Cochran R, Croessmann S, Zabransky DJ, Wong HY, Toro PV, Cidado J, Blair BG (2014). Detection of cancer DNA in plasma of patients with early-stage breast cancer. Clin Cancer Res.

[12] Mirza S, Sharma G, Parshad R, Srivastava A, Gupta SD, Ralhan R (2012). Clinical significance of promoter hypermethylation of ERbeta and RARbeta2 in tumor and serum DNA in Indian breast cancer patients. Ann Surg Oncol.

[13] Gasch C, Oldopp T, Mauermann O, Gorges TM, Andreas A, Coith C, Muller V, Fehm T, Janni W, Pantel K, Riethdorf S (2016). Frequent detection of PIK3CA mutations in single circulating tumor cells of patients suffering from HER2-negative metastatic breast cancer. Mol Oncol.

[14] Ulz P, Thallinger GG, Auer M, Graf R, Kashofer K, Jahn SW, Abete L, Pristauz G, Petru E, Geigl JB (2016). Inferring expressed genes by whole-genome sequencing of plasma DNA. Nat Genet.

[15] Guo Z, Yang F, Zhang J, Zhang Z, Li K, Tian Q, Hou H, Xu C, Lu Q, Ren Z (2020). Whole-genome promoter profiling of plasma DNA exhibits diagnostic value for placenta-origin pregnancy complications. Adv Sci.

[16] Snyder MW, Kircher M, Hill AJ, Daza RM, Shendure J (2016). Cell-free DNA comprises an in vivo nucleosome footprint that informs its tissues-of-origin. Cell.

[17] Ali HR, Chlon L, Pharoah PD, Markowetz F, Caldas C (2016). Patterns of immune infiltration in breast cancer and their clinical implications: a gene-expression-based retrospective study. PLoS Med.

[18] Foulds GA, Vadakekolathu J, Abdel-Fatah TMA, Nagarajan D, Reeder S, Johnson C, Hood S, Moseley PM, Chan SYT, Pockley AG (2028). Immune-phenotyping and transcriptomic profiling of peripheral blood mononuclear cells from patients with breast cancer: identification of a 3 gene signature which predicts relapse of triple negative breast cancer. Front Immunol.

[19] Li H, Handsaker B, Wysoker A, Fennell T, Ruan J, Homer N, Marth G, Abecasis G, Durbin R, Genome Project Data Processing S (2009). The sequence alignment/map format and SAMtools. Bioinformatics.

[20] Adalsteinsson VA, Ha G, Freeman SS, Choudhury AD, Stover DG, Parsons HA, Gydush G, Reed SC, Rotem D, Rhoades J (2017). Scalable whole-exome sequencing of cell-free DNA reveals high concordance with metastatic tumors. Nat Commun.

[21] Guo ZW, Xiao WW, Yang XX, Yang X, Cai GX, Wang XJ, Han BW, Li K, Zhai XM, Li FX (2020). Noninvasive prediction of response to cancer therapy using promoter profiling of circulating cell-free DNA. Clin Transl Med.

[22] Casper J, Zweig AS, Villarreal C, Tyner C, Speir ML, Rosenbloom KR, Raney BJ, Lee CM, Lee BT, Karolchik D (2018). The UCSC genome browser database: 2018 update. Nucleic Acids Res.

[23] Chen K, Xi Y, Pan X, Li Z, Kaestner K, Tyler J, Dent S, He X, Li W (2013). DANPOS: dynamic analysis of nucleosome position and occupancy by sequencing. Genome Res.

[24] Robin X, Turck N, Hainard A, Tiberti N, Lisacek F, Sanchez JC, Muller M (2011). pROC: an open-source package for R and S+ to analyze and compare ROC curves. BMC Bioinformatics.

[25] Zhou Y, Zhou B, Pache L, Chang M, Khodabakhshi AH, Tanaseichuk O, Benner C, Chanda SK (2019). Metascape provides a biologist-oriented resource for the analysis of systems-level datasets. Nat Commun.

[26] Ahmed AR (2016). HER2 expression is a strong independent predictor of nodal metastasis in breast cancer. J Egypt Natl Canc Inst.

[27] Van Calster B, Vanden Bempt I, Drijkoningen M, Pochet N, Cheng J, Van Huffel S, Hendrickx W, Decock J, Huang HJ, Leunen K (2009). Axillary lymph node status of operable breast cancers by combined steroid receptor and HER-2 status: triple positive tumours are more likely lymph node positive. Breast Cancer Res Treat.

[28] Shibata A, Nagaya T, Imai T, Funahashi H, Nakao A, Seo H (2002). Inhibition of NF-kappaB activity decreases the VEGF mRNA expression in MDA-MB-231 breast cancer cells. Breast Cancer Res Treat.

[29] Wang J, Li S, Li X, Li B, Li Y, Xia K, Yang Y, Aman S, Wang M, Wu H (2019). Circadian protein BMAL1 promotes breast cancer cell invasion and metastasis by up-regulating matrix metalloproteinase9 expression. Cancer Cell Int.

[30] Sethuraman A, Brown M, Krutilina R, Wu ZH, Seagroves TN, Pfeffer LM, Fan M (2018). BHLHE40 confers a pro-survival and pro-metastatic phenotype to breast cancer cells by modulating HBEGF secretion. Breast Cancer Res.

[31] Zheng Q, Wang C, Wang L, Zhang D, Liu N, Ming X, Zhou H, Guli Q, Liu Y (2018). Interaction with SP1, but not binding to the E-box motifs, is responsible for BHLHE40/DEC1-induced transcriptional suppression of CLDN1 and cell invasion in MCF-7 cells. Mol Carcinog.

[32] Li W, Shen M, Jiang YZ, Zhang R, Zheng H, Wei Y, Shao ZM, Kang Y (2020). Deubiquitinase USP20 promotes breast cancer metastasis by stabilizing SNAI2. Genes Dev.

[33] Ulz P, Perakis S, Zhou Q, Moser T, Belic J, Lazzeri I, Wolfler A, Zebisch A, Gerger A, Pristauz G (2019). Inference of transcription factor binding from cell-free DNA enables tumor subtype prediction and early detection. Nat Commun.

