# Supplementary material for: Noninvasive prediction of axillary lymph node status in breast cancer using promoter profiling of circulating cell-free DNA
Source: J Transl Med. 2022 Dec 3;20:557. doi: 10.1186/s12967-022-03724-w (PMC9719247; doi:10.1186/s12967-022-03724-w)
Supplement: Supplementary file 1 — Additional file 1: Figure S1. The tumor DNA fraction between ALN-positive and ALN-negative patients. Table S1. Genes with differential promoter coverage between ALN-positive and -negative groups. Table S2. The genes in PPCNM. Table S3. The predictive efficacy of PPCNM in different tumor fraction. Table S4. Predictive efficacy of the PPCNM with characteristics. [file 12967_2022_3724_MOESM1_ESM.docx]

**Additional file 1**

**Additional Materials**

| **Additional Figures and Tables** |
| --- |
| Figure S1. The tumor DNA fraction between ALN-positive and ALN-negative patients |
| Table S1. Genes with differential promoter coverage between ALN-positive and -negative groups |
| Table S2. The genes in PPCNM |
| Table S3. The predictive efficacy of PPCNM in different tumor fraction |
| Table S4. Predictive efficacy of the PPCNM with characteristics |

**Figure S1. Tumor DNA fraction between ALN-positive and ALN-negative groups.**


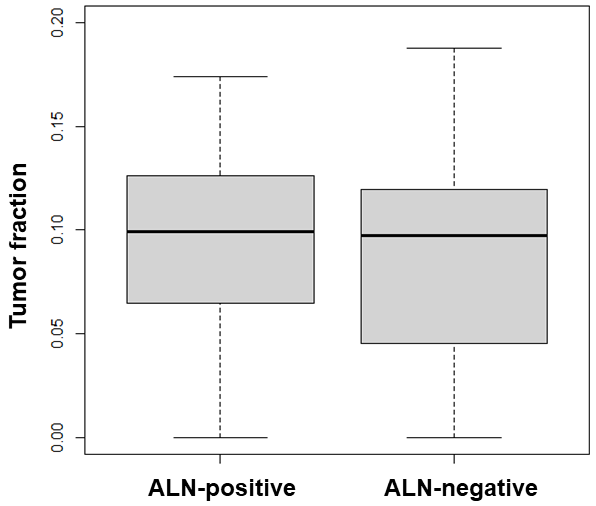


**Table S1. Genes with differential promoter coverage between ALN-positive and -negative groups**

| RefSeq_ID | Region | FC | FDR |
| --- | --- | --- | --- |
| NM_001077494 | chr10.104153334.104155334 | 0.553092 | 0.042396 |
| NM_024326 | chr10.104178570.104180570 | 1.703252 | 0.04334 |
| NM_001270965 | chr10.104178691.104180691 | 1.643594 | 0.042047 |
| NM_001351189 | chr10.104935814.104937814 | 1.753981 | 0.040468 |
| NM_001272013 | chr10.106097251.106099251 | 1.771122 | 0.042396 |
| NM_016824 | chr10.111766693.111768693 | 0.512202 | 0.038643 |
| NM_001244949 | chr10.113942537.113944537 | 1.713593 | 0.038643 |
| NM_182601 | chr10.115523577.115525577 | 1.584535 | 0.045577 |
| NM_001195608 | chr10.124144584.124146584 | 2.27123 | 0.034724 |
| NM_004406 | chr10.124319180.124321180 | 1.680628 | 0.034724 |
| NM_032182 | chr10.126489353.126491353 | 0.561607 | 0.045577 |
| NM_018180 | chr10.127568884.127570884 | 1.703607 | 0.041292 |
| NM_001004298 | chr10.128209062.128211062 | 1.615585 | 0.042396 |
| NM_001355042 | chr10.131908085.131910085 | 0.438693 | 0.036645 |
| NM_004052 | chr10.133794517.133796517 | 0.624939 | 0.046944 |
| NM_001318337 | chr10.14013369.14015369 | 1.660425 | 0.036645 |
| NM_001320740 | chr10.14579541.14581541 | 0.573417 | 0.038643 |
| NM_001039844 | chr10.15129775.15131775 | 2.3584 | 0.038643 |
| NM_014241 | chr10.17658373.17660373 | 0.506788 | 0.034724 |
| NM_201570 | chr10.18688512.18690512 | 1.630433 | 0.036749 |
| NM_001351118 | chr10.18939566.18941566 | 1.572984 | 0.04535 |
| NM_001282736 | chr10.20104371.20106371 | 1.715941 | 0.042047 |
| NM_001173484 | chr10.21462116.21464116 | 0.590101 | 0.045577 |
| NM_001282767 | chr10.24496719.24498719 | 1.511972 | 0.046944 |
| NM_012342 | chr10.28965423.28967423 | 0.581806 | 0.046944 |
| NM_018109 | chr10.30637267.30639267 | 1.534045 | 0.04535 |
| NM_001320961 | chr10.30722563.30724563 | 1.830512 | 0.046944 |
| NM_019619 | chr10.35103253.35105253 | 0.465629 | 0.041292 |
| NM_001098208 | chr10.43891279.43893279 | 0.639263 | 0.042396 |
| NM_001137549 | chr10.47180688.47182688 | 1.735033 | 0.034724 |
| NM_001031746 | chr10.50322577.50324577 | 0.559205 | 0.045577 |
| NM_001253909 | chr10.5135567.5137567 | 2.243659 | 0.034724 |
| NM_001142772 | chr10.56560051.56562051 | 2.096048 | 0.038643 |
| NM_001351095 | chr10.6019704.6021704 | 1.840804 | 0.042047 |
| NM_001242413 | chr10.6621263.6623263 | 0.43934 | 0.038643 |
| NM_001323265 | chr10.6621608.6623608 | 0.42622 | 0.040468 |
| NM_015634 | chr10.70747476.70749476 | 1.626821 | 0.045577 |
| NM_001322367 | chr10.71077602.71079602 | 0.414102 | 0.034724 |
| NM_032797 | chr10.71891690.71893690 | 1.735444 | 0.038643 |
| NM_024875 | chr10.75409797.75411797 | 1.729447 | 0.036269 |
| NM_016364 | chr10.76858248.76860248 | 0.642455 | 0.041292 |
| NM_001174156 | chr10.76870392.76872392 | 1.616128 | 0.036749 |
| NM_001080518 | chr10.90483300.90485300 | 1.699996 | 0.045577 |
| NM_016195 | chr10.91460346.91462346 | 1.608631 | 0.040468 |
| NM_001319194 | chr10.94589934.94591934 | 1.777844 | 0.040468 |
| NM_001198853 | chr10.96828254.96830254 | 1.555711 | 0.042047 |
| NM_001256423 | chr10.99029447.99031447 | 1.89093 | 0.038643 |
| NM_032900 | chr10.99051430.99053430 | 0.6372 | 0.036749 |
| NM_003015 | chr10.99530756.99532756 | 0.499426 | 0.041292 |
| NM_000646 | chr1.100315530.100317530 | 2.15326 | 0.034724 |
| NM_001205252 | chr1.1008687.1010687 | 1.607348 | 0.042047 |
| NM_007269 | chr1.109288256.109290256 | 2.142084 | 0.044041 |
| NM_001040709 | chr1.110008099.110010099 | 1.58891 | 0.04535 |
| NM_001165 | chr11.102187180.102189180 | 1.719336 | 0.044041 |
| NM_022122 | chr11.102575468.102577468 | 0.626524 | 0.044041 |
| NM_033135 | chr11.104034027.104036027 | 1.635602 | 0.034724 |
| NM_001257118 | chr11.104904884.104906884 | 0.642103 | 0.044041 |
| NM_001318750 | chr11.105892014.105894014 | 0.54779 | 0.038543 |
| NM_001010898 | chr1.110692135.110694135 | 0.624388 | 0.04535 |
| NM_152587 | chr11.108337258.108339258 | 1.66225 | 0.036645 |
| NM_001201545 | chr1.110880944.110882944 | 0.568645 | 0.045577 |
| NM_033390 | chr11.109963086.109965086 | 0.618004 | 0.045577 |
| NM_178510 | chr11.113257512.113259512 | 0.615326 | 0.036749 |
| NM_001018011 | chr11.113930228.113932228 | 0.605249 | 0.045577 |
| NM_001320638 | chr1.111414721.111416721 | 1.799003 | 0.036749 |
| NM_020693 | chr11.117666976.117668976 | 0.619953 | 0.042396 |
| NM_001258209 | chr11.118954586.118956586 | 1.884425 | 0.04334 |
| NM_001282358 | chr11.119038042.119040042 | 1.552638 | 0.036749 |
| NM_001001965 | chr11.123809323.123811323 | 0.663836 | 0.042047 |
| NM_139055 | chr11.130317868.130319868 | 0.63947 | 0.045577 |
| NM_001318514 | chr11.1323020.1325020 | 0.605169 | 0.041292 |
| NM_001330350 | chr1.11332254.11334254 | 1.529321 | 0.045577 |
| NM_032228 | chr11.13689205.13691205 | 1.917011 | 0.042396 |
| NM_004195 | chr1.1141089.1143089 | 0.660072 | 0.041292 |
| NM_001177314 | chr11.14385052.14387052 | 0.594197 | 0.038643 |
| NM_205848 | chr1.114694062.114696062 | 1.577258 | 0.042396 |
| NM_001741 | chr11.14992832.14994832 | 1.648591 | 0.034724 |
| NM_000728 | chr11.15094145.15096145 | 1.880781 | 0.034724 |
| NM_001160234 | chr1.116924991.116926991 | 1.666726 | 0.034724 |
| NM_199293 | chr11.2192035.2194035 | 0.634126 | 0.038543 |
| NM_020346 | chr11.22358666.22360666 | 1.76251 | 0.045577 |
| NM_181798 | chr11.2481683.2483683 | 0.657351 | 0.046944 |
| NM_001143807 | chr11.27740294.27742294 | 1.781527 | 0.042396 |
| NM_023014 | chr1.12915940.12917940 | 1.525083 | 0.04535 |
| NM_003641 | chr11.312990.314990 | 1.67731 | 0.034724 |
| NM_001258465 | chr11.31831690.31833690 | 1.790034 | 0.040468 |
| NM_001604 | chr11.31831901.31833901 | 1.765095 | 0.041292 |
| NM_001258463 | chr11.31832731.31834731 | 1.688341 | 0.044041 |
| NM_024081 | chr11.32850480.32852480 | 2.022949 | 0.034724 |
| NM_001076786 | chr11.32913723.32915723 | 0.599306 | 0.045577 |
| NM_001350497 | chr1.1332722.1334722 | 0.570119 | 0.041292 |
| NM_001278162 | chr11.33278876.33280876 | 0.569383 | 0.048993 |
| NM_001024661 | chr1.13672511.13674511 | 2.208453 | 0.036645 |
| NM_001665 | chr11.3861213.3863213 | 1.746575 | 0.046944 |
| NM_001303029 | chr11.391598.393598 | 0.637794 | 0.042047 |
| NM_014455 | chr1.145609989.145611989 | 0.543129 | 0.038643 |
| NM_003654 | chr11.45686206.45688206 | 2.173391 | 0.038643 |
| NM_001005170 | chr11.4607020.4609020 | 1.683091 | 0.034724 |
| NM_001105540 | chr11.46382144.46384144 | 1.566854 | 0.046944 |
| NM_001184975 | chr11.47206432.47208432 | 0.545161 | 0.042396 |
| NM_001123375 | chr1.149784236.149786236 | 2.167515 | 0.036749 |
| NM_032132 | chr1.150692364.150694364 | 1.592093 | 0.045577 |
| NM_001134939 | chr1.151734444.151736444 | 1.734643 | 0.034724 |
| NM_001301371 | chr1.151734955.151736955 | 1.968027 | 0.034724 |
| NM_014624 | chr1.153507717.153509717 | 0.547889 | 0.034724 |
| NM_001255980 | chr1.153939314.153941314 | 1.602902 | 0.04334 |
| NM_001271960 | chr1.153939660.153941660 | 1.571795 | 0.038543 |
| NM_001255979 | chr1.153939674.153941674 | 1.52228 | 0.042047 |
| NM_001826 | chr1.154946117.154948117 | 0.595788 | 0.044041 |
| NM_002455 | chr1.155177489.155179489 | 1.70942 | 0.042047 |
| NM_001171812 | chr1.155210066.155212066 | 1.948402 | 0.034724 |
| NM_000157 | chr1.155210069.155212069 | 1.948402 | 0.034724 |
| NM_018489 | chr1.155531324.155533324 | 1.739003 | 0.04535 |
| NM_001005491 | chr11.55734939.55736939 | 1.739641 | 0.036645 |
| NM_001004058 | chr11.55926793.55928793 | 1.880723 | 0.036645 |
| NM_003145 | chr1.155989758.155991758 | 1.60049 | 0.045577 |
| NM_001004746 | chr11.55999661.56001661 | 1.765428 | 0.034724 |
| NM_001320455 | chr1.156390558.156392558 | 1.987202 | 0.040468 |
| NM_033396 | chr11.57091413.57093413 | 0.552442 | 0.041292 |
| NM_001032295 | chr11.57364704.57366704 | 2.163431 | 0.034724 |
| NM_003126 | chr1.158655505.158657505 | 1.978205 | 0.039622 |
| NM_002432 | chr1.158800092.158802092 | 1.815043 | 0.04334 |
| NM_001320010 | chr1.158969102.158971102 | 1.850484 | 0.034724 |
| NM_001005324 | chr11.59480318.59482318 | 1.534912 | 0.042396 |
| NM_001001917 | chr11.6047971.6049971 | 1.670723 | 0.039622 |
| NM_152366 | chr1.161067180.161069180 | 1.572268 | 0.041292 |
| NM_004550 | chr1.161168104.161170104 | 0.606186 | 0.046944 |
| NM_001145077 | chr11.61275271.61277271 | 0.622763 | 0.038543 |
| NM_001329120 | chr1.161519527.161521527 | 0.652725 | 0.038643 |
| NM_004265 | chr11.61594504.61596504 | 0.483859 | 0.048389 |
| NM_001346446 | chr11.62312159.62314159 | 0.393042 | 0.032921 |
| NM_001346445 | chr11.62312238.62314238 | 0.405677 | 0.032921 |
| NM_031264 | chr11.624067.626067 | 0.644904 | 0.042047 |
| NM_001043229 | chr11.62431778.62433778 | 2.763933 | 0.036269 |
| NM_001081491 | chr11.62571964.62573964 | 1.534826 | 0.04535 |
| NM_001307977 | chr11.62606131.62608131 | 1.922845 | 0.039622 |
| NM_001170726 | chr11.64050810.64052810 | 1.680106 | 0.040468 |
| NM_032989 | chr11.64051176.64053176 | 1.648062 | 0.049794 |
| NM_001300802 | chr11.64125624.64127624 | 0.500928 | 0.036749 |
| NM_138734 | chr11.64409787.64411787 | 0.378448 | 0.034724 |
| NM_001278250 | chr11.65148857.65150857 | 1.688547 | 0.04535 |
| NM_198897 | chr11.65655010.65657010 | 2.068136 | 0.034724 |
| NM_020470 | chr11.66055638.66057638 | 0.662144 | 0.045577 |
| NM_005700 | chr11.66246483.66248483 | 1.505984 | 0.042396 |
| NM_018994 | chr1.16677986.16679986 | 1.943489 | 0.034724 |
| NM_001243224 | chr11.67159775.67161775 | 0.626128 | 0.046944 |
| NM_001130848 | chr11.67271843.67273843 | 0.480152 | 0.036645 |
| NM_001322924 | chr1.167598429.167600429 | 2.147703 | 0.038543 |
| NM_052862 | chr1.167598473.167600473 | 2.143161 | 0.042047 |
| NM_001145278 | chr1.16766166.16768166 | 1.547826 | 0.039622 |
| NM_001267614 | chr1.168105811.168107811 | 1.599815 | 0.038643 |
| NM_004923 | chr11.68517988.68519988 | 0.590396 | 0.04334 |
| NM_001300968 | chr1.169428907.169430907 | 1.770155 | 0.038543 |
| NM_012309 | chr11.70934842.70936842 | 1.604123 | 0.036749 |
| NM_006894 | chr1.171059017.171061017 | 1.734507 | 0.034724 |
| NM_002022 | chr1.171282321.171284321 | 0.599535 | 0.040468 |
| NM_005699 | chr11.71709108.71711108 | 0.635942 | 0.038643 |
| NM_017907 | chr11.71813433.71815433 | 0.63101 | 0.046944 |
| NM_001282750 | chr1.172501257.172503257 | 1.590397 | 0.039622 |
| NM_175733 | chr11.7272180.7274180 | 0.646149 | 0.039622 |
| NM_153614 | chr11.73660363.73662363 | 0.550147 | 0.040468 |
| NM_001300851 | chr1.173961210.173963210 | 1.515602 | 0.04535 |
| NM_001235 | chr11.75272100.75274100 | 1.534673 | 0.044041 |
| NM_001302826 | chr11.7694474.7696474 | 1.505752 | 0.042396 |
| NM_173039 | chr11.77299679.77301679 | 0.621078 | 0.045577 |
| NM_001318824 | chr11.776502.778502 | 0.244016 | 0.034724 |
| NM_012296 | chr11.78051926.78053926 | 1.587737 | 0.039622 |
| NM_001324310 | chr1.178993938.178995938 | 1.593712 | 0.04334 |
| NM_002065 | chr1.182360341.182362341 | 1.815272 | 0.034724 |
| NM_033345 | chr1.182641067.182643067 | 1.650086 | 0.036749 |
| NM_018891 | chr1.183154173.183156173 | 1.644453 | 0.036269 |
| NM_005717 | chr1.183604076.183606076 | 3.303561 | 0.032921 |
| NM_015149 | chr1.183604181.183606181 | 2.958519 | 0.032921 |
| NM_178545 | chr1.1849740.1851740 | 0.626994 | 0.044041 |
| NM_173556 | chr11.85565143.85567143 | 0.589772 | 0.040468 |
| NM_001127709 | chr1.186264404.186266404 | 2.034609 | 0.038643 |
| NM_001164246 | chr1.186343889.186345889 | 1.550532 | 0.046944 |
| NM_022576 | chr1.186416856.186418856 | 1.983625 | 0.036749 |
| NM_001286094 | chr11.8985320.8987320 | 1.725731 | 0.036749 |
| NM_020974 | chr11.9112150.9114150 | 0.619661 | 0.038643 |
| NM_001330199 | chr11.9112154.9114154 | 0.619661 | 0.038643 |
| NM_001286139 | chr11.92929621.92931621 | 1.933238 | 0.038643 |
| NM_024529 | chr1.193090087.193092087 | 2.106276 | 0.036749 |
| NM_015012 | chr11.9335315.9337315 | 1.931211 | 0.046944 |
| NM_015368 | chr11.93861093.93863093 | 0.598559 | 0.038643 |
| NM_001994 | chr1.197035397.197037397 | 1.814797 | 0.036645 |
| NM_080921 | chr1.198607097.198609097 | 1.673257 | 0.044041 |
| NM_001278164 | chr1.19968722.19970722 | 0.624673 | 0.039622 |
| NM_005380 | chr1.19969666.19971666 | 0.522687 | 0.04334 |
| NM_005298 | chr1.200841082.200843082 | 0.542988 | 0.041292 |
| NM_001193570 | chr1.201475387.201477387 | 0.609748 | 0.036645 |
| NM_018085 | chr1.201797287.201799287 | 1.565291 | 0.034724 |
| NM_001017403 | chr1.202162117.202164117 | 1.617235 | 0.04535 |
| NM_014589 | chr1.20249110.20251110 | 1.829466 | 0.034724 |
| NM_001174108 | chr1.203765650.203767650 | 1.533292 | 0.045577 |
| NM_001297761 | chr1.204120310.204122310 | 0.507975 | 0.036749 |
| NM_203376 | chr1.205052588.205054588 | 1.621197 | 0.040468 |
| NM_001877 | chr1.207626644.207628644 | 1.660289 | 0.038643 |
| NM_022496 | chr12.100592864.100594864 | 2.121681 | 0.040468 |
| NM_207375 | chr12.10083980.10085980 | 1.630179 | 0.042396 |
| NM_003211 | chr12.104358592.104360592 | 0.544221 | 0.044041 |
| NM_001351062 | chr12.10457758.10459758 | 1.730975 | 0.045577 |
| NM_002260 | chr12.10587592.10589592 | 1.565053 | 0.046944 |
| NM_002259 | chr12.10604979.10606979 | 2.703854 | 0.032921 |
| NM_213658 | chr12.10606215.10608215 | 1.924747 | 0.034724 |
| NM_001304448 | chr12.10606284.10608284 | 1.798223 | 0.034724 |
| NM_023917 | chr12.10961767.10963767 | 1.622652 | 0.034724 |
| NM_016301 | chr12.110905089.110907089 | 0.581041 | 0.045577 |
| NM_152591 | chr12.111283763.111285763 | 1.799482 | 0.041292 |
| NM_015267 | chr12.111470827.111472827 | 0.631904 | 0.04535 |
| NM_176885 | chr12.11183006.11185006 | 1.51258 | 0.034724 |
| NM_001320141 | chr12.112855647.112857647 | 0.587602 | 0.042396 |
| NM_080601 | chr12.112855701.112857701 | 0.601974 | 0.04334 |
| NM_001204218 | chr12.117798607.117800607 | 0.634834 | 0.039622 |
| NM_001346815 | chr12.118453465.118455465 | 0.648302 | 0.046944 |
| NM_019086 | chr12.118540810.118542810 | 0.551196 | 0.042396 |
| NM_015918 | chr12.121018197.121020197 | 0.639321 | 0.042047 |
| NM_198202 | chr12.121018201.121020201 | 0.639321 | 0.042047 |
| NM_001033677 | chr12.121077421.121079421 | 1.52253 | 0.04535 |
| NM_016237 | chr12.121789265.121791265 | 1.760104 | 0.034724 |
| NM_152269 | chr12.123716843.123718843 | 1.682754 | 0.038643 |
| NM_001194995 | chr12.123717027.123719027 | 1.747452 | 0.038543 |
| NM_001310 | chr12.12763766.12765766 | 0.545351 | 0.04535 |
| NM_001136103 | chr12.128750947.128752947 | 0.504164 | 0.034724 |
| NM_007197 | chr12.130646003.130648003 | 0.664955 | 0.041292 |
| NM_001349671 | chr1.213223574.213225574 | 1.745546 | 0.034724 |
| NM_016155 | chr12.132311937.132313937 | 0.43568 | 0.040468 |
| NM_015409 | chr12.132433464.132435464 | 0.526239 | 0.049794 |
| NM_001300776 | chr12.133655788.133657788 | 0.630688 | 0.04334 |
| NM_001191054 | chr12.133811422.133813422 | 0.521902 | 0.036749 |
| NM_003760 | chr1.21436876.21438876 | 1.501773 | 0.041292 |
| NM_001198803 | chr1.21493532.21495532 | 1.521054 | 0.04334 |
| NM_001243610 | chr12.16758734.16760734 | 1.705699 | 0.04334 |
| NM_138796 | chr1.217803665.217805665 | 1.697643 | 0.034724 |
| NM_019844 | chr12.20962637.20964637 | 1.643562 | 0.044041 |
| NM_001009562 | chr12.21167629.21169629 | 1.826965 | 0.038643 |
| NM_001324080 | chr1.222912025.222914025 | 1.802887 | 0.04535 |
| NM_005747 | chr1.22327148.22329148 | 1.602387 | 0.036645 |
| NM_001261415 | chr12.24102966.24104966 | 1.548788 | 0.04334 |
| NM_002296 | chr1.225614815.225616815 | 3.716619 | 0.034724 |
| NM_001164746 | chr12.26204490.26206490 | 1.548675 | 0.038643 |
| NM_030762 | chr12.26277003.26279003 | 0.664658 | 0.044041 |
| NM_031944 | chr1.226410318.226412318 | 1.559247 | 0.040468 |
| NM_002221 | chr1.226925876.226927876 | 0.50416 | 0.045577 |
| NM_001658 | chr1.228269360.228271360 | 1.785522 | 0.039622 |
| NM_014409 | chr1.229760794.229762794 | 1.949068 | 0.038543 |
| NM_014777 | chr1.229760962.229762962 | 1.92524 | 0.039622 |
| NM_001193451 | chr12.29935743.29937743 | 0.540876 | 0.036749 |
| NM_001164537 | chr1.231761560.231763560 | 0.538047 | 0.034724 |
| NM_001286522 | chr12.3981614.3983614 | 1.664044 | 0.04535 |
| NM_001031748 | chr12.40018968.40020968 | 1.726169 | 0.036749 |
| NM_001142679 | chr12.45608769.45610769 | 0.543246 | 0.04334 |
| NM_001143824 | chr12.47218780.47220780 | 1.686673 | 0.034724 |
| NM_001004691 | chr1.248486870.248488870 | 1.617883 | 0.034724 |
| NM_001322464 | chr1.249119484.249121484 | 0.625268 | 0.04535 |
| NM_198900 | chr12.50100197.50102197 | 0.573684 | 0.045577 |
| NM_175834 | chr12.53227077.53229077 | 0.595687 | 0.036749 |
| NM_170754 | chr12.53442834.53444834 | 0.658718 | 0.045577 |
| NM_017410 | chr12.54331575.54333575 | 0.643246 | 0.042396 |
| NM_001127322 | chr12.54652329.54654329 | 1.786739 | 0.048993 |
| NM_001127321 | chr12.54652370.54654370 | 1.827694 | 0.044041 |
| NM_001005183 | chr12.55819037.55821037 | 1.958216 | 0.045577 |
| NM_014182 | chr12.56210805.56212805 | 1.793454 | 0.038643 |
| NM_001345 | chr12.56323945.56325945 | 0.461988 | 0.036749 |
| NM_194359 | chr12.56614753.56616753 | 1.859763 | 0.038643 |
| NM_005981 | chr12.58137771.58139771 | 1.780253 | 0.034724 |
| NM_153377 | chr12.59313319.59315319 | 0.665737 | 0.042047 |
| NM_001278596 | chr12.6053425.6055425 | 1.544994 | 0.042047 |
| NM_024037 | chr1.26184949.26186949 | 1.551045 | 0.04535 |
| NM_002076 | chr12.65152226.65154226 | 1.742651 | 0.045577 |
| NM_001330187 | chr12.65152276.65154276 | 1.742879 | 0.045577 |
| NM_001300918 | chr12.66217239.66219239 | 0.535065 | 0.04334 |
| NM_001205029 | chr12.68725194.68727194 | 1.742729 | 0.044041 |
| NM_021969 | chr1.27239567.27241567 | 0.608456 | 0.042396 |
| NM_013381 | chr12.72665528.72667528 | 0.459476 | 0.034724 |
| NM_001329454 | chr12.7281610.7283610 | 1.567841 | 0.036749 |
| NM_152365 | chr1.27285901.27287901 | 0.598181 | 0.036749 |
| NM_001351124 | chr12.7340280.7342280 | 0.563739 | 0.036749 |
| NM_001131026 | chr12.7340758.7342758 | 0.593877 | 0.036749 |
| NM_001294346 | chr12.751851.753851 | 0.455622 | 0.034724 |
| NM_001355024 | chr12.75723112.75725112 | 1.844356 | 0.044041 |
| NM_001355033 | chr12.75783717.75785717 | 1.801517 | 0.042047 |
| NM_152436 | chr12.75783849.75785849 | 1.761169 | 0.042047 |
| NM_006851 | chr12.75873512.75875512 | 1.640456 | 0.036749 |
| NM_004244 | chr12.7655414.7657414 | 1.845277 | 0.038543 |
| NM_001244992 | chr12.80327731.80329731 | 2.315302 | 0.042396 |
| NM_001100917 | chr12.85429055.85431055 | 2.149983 | 0.034724 |
| NM_001079910 | chr12.85429098.85431098 | 2.114651 | 0.034724 |
| NM_006183 | chr12.86267072.86269072 | 1.748411 | 0.038643 |
| NM_001282424 | chr12.8996599.8998599 | 1.546679 | 0.04535 |
| NM_007035 | chr12.91451131.91453131 | 2.06815 | 0.034724 |
| NM_001301024 | chr12.93771325.93773325 | 1.681563 | 0.036269 |
| NM_001204414 | chr1.32041085.32043085 | 0.658612 | 0.045577 |
| NM_001135816 | chr13.24462577.24464577 | 1.699564 | 0.036645 |
| NM_005932 | chr13.24462587.24464587 | 1.721885 | 0.036645 |
| NM_001348114 | chr13.24462743.24464743 | 1.54399 | 0.04535 |
| NM_001319677 | chr1.32536631.32538631 | 1.591094 | 0.04334 |
| NM_012316 | chr1.32572643.32574643 | 1.72448 | 0.044041 |
| NM_001321534 | chr13.31207315.31209315 | 0.510816 | 0.036645 |
| NM_001349704 | chr13.31735158.31737158 | 0.62522 | 0.045577 |
| NM_001142296 | chr13.36919646.36921646 | 2.076483 | 0.041292 |
| NM_152903 | chr13.41705936.41707936 | 0.622685 | 0.039622 |
| NM_001352984 | chr1.34328677.34330677 | 1.974662 | 0.034724 |
| NM_001127615 | chr13.44202613.44204613 | 1.712277 | 0.035608 |
| NM_001286763 | chr13.46960635.46962635 | 1.594884 | 0.046944 |
| NM_001164211 | chr13.47126295.47128295 | 0.469721 | 0.038643 |
| NM_001162498 | chr13.48986653.48988653 | 1.539678 | 0.04535 |
| NM_001162497 | chr13.49000043.49002043 | 1.587786 | 0.044041 |
| NM_030911 | chr13.49821046.49823046 | 0.505849 | 0.034724 |
| NM_001287337 | chr13.50017262.50019262 | 0.534394 | 0.038643 |
| NM_001320727 | chr13.50017428.50019428 | 0.548588 | 0.034724 |
| NM_001282169 | chr13.53023379.53025379 | 1.67822 | 0.042396 |
| NM_178548 | chr1.36037970.36039970 | 0.541373 | 0.044041 |
| NM_001190481 | chr1.36234551.36236551 | 0.563185 | 0.036749 |
| NM_014466 | chr1.36548675.36550675 | 1.650018 | 0.045577 |
| NM_152492 | chr1.3667964.3669964 | 1.536359 | 0.038643 |
| NM_001162530 | chr1.36770993.36772993 | 0.544355 | 0.034724 |
| NM_024676 | chr1.36771707.36773707 | 0.659291 | 0.046944 |
| NM_203487 | chr13.67803468.67805468 | 1.510369 | 0.046944 |
| NM_032881 | chr1.36862560.36864560 | 1.950995 | 0.044041 |
| NM_001287394 | chr13.76122575.76124575 | 0.497496 | 0.035608 |
| NM_001270952 | chr13.76122615.76124615 | 0.516032 | 0.038643 |
| NM_006002 | chr13.76122885.76124885 | 0.497026 | 0.038643 |
| NM_207356 | chr1.3815857.3817857 | 0.533448 | 0.036645 |
| NM_032229 | chr13.86372483.86374483 | 1.729425 | 0.036645 |
| NM_015567 | chr13.88323869.88325869 | 0.294821 | 0.034724 |
| NM_016337 | chr14.100530750.100532750 | 1.602404 | 0.035608 |
| NM_001159531 | chr14.101033407.101035407 | 0.48215 | 0.036645 |
| NM_001352915 | chr14.102227706.102229706 | 2.305049 | 0.034724 |
| NM_199165 | chr14.105195180.105197180 | 0.642108 | 0.036749 |
| NM_001243127 | chr14.105766147.105768147 | 0.46727 | 0.042396 |
| NM_001519 | chr14.105766329.105768329 | 0.405051 | 0.044041 |
| NM_002935 | chr14.21358561.21360561 | 1.604082 | 0.04334 |
| NM_001354569 | chr14.21493469.21495469 | 1.643364 | 0.042047 |
| NM_017815 | chr14.23425351.23427351 | 0.639685 | 0.042396 |
| NM_017924 | chr14.23563682.23565682 | 0.62731 | 0.045577 |
| NM_020834 | chr14.23754309.23756309 | 1.75974 | 0.036645 |
| NM_001354677 | chr14.24035722.24037722 | 0.567514 | 0.034724 |
| NM_001282475 | chr14.24035733.24037733 | 0.550743 | 0.034724 |
| NM_001354673 | chr14.24035997.24037997 | 0.533028 | 0.035608 |
| NM_001282474 | chr14.24036045.24038045 | 0.583867 | 0.036749 |
| NM_001318835 | chr14.24098323.24100323 | 1.848828 | 0.034724 |
| NM_198083 | chr14.24457026.24459026 | 0.598516 | 0.042047 |
| NM_015473 | chr14.31888788.31890788 | 0.513303 | 0.04535 |
| NM_001308110 | chr14.36788882.36790882 | 1.593144 | 0.045577 |
| NM_001349127 | chr1.44456267.44458267 | 0.639523 | 0.044041 |
| NM_001349128 | chr1.44456518.44458518 | 0.660532 | 0.042396 |
| NM_001308112 | chr14.45429335.45431335 | 1.949659 | 0.045577 |
| NM_015091 | chr14.45430392.45432392 | 1.953239 | 0.038543 |
| NM_001030001 | chr14.50052134.50054134 | 2.076838 | 0.038643 |
| NM_006575 | chr14.50998376.51000376 | 0.388683 | 0.036749 |
| NM_001127713 | chr14.50998799.51000799 | 0.461411 | 0.038643 |
| NM_021818 | chr14.51134071.51136071 | 1.74344 | 0.036749 |
| NM_014584 | chr14.53161649.53163649 | 0.624426 | 0.041292 |
| NM_001130701 | chr14.53195882.53197882 | 1.702852 | 0.042396 |
| NM_001166588 | chr1.45451394.45453394 | 1.618722 | 0.038643 |
| NM_024602 | chr1.45476027.45478027 | 0.566059 | 0.045577 |
| NM_174978 | chr14.60951764.60953764 | 1.812233 | 0.036749 |
| NM_001243084 | chr14.62163339.62165339 | 2.245077 | 0.034724 |
| NM_145171 | chr14.63784593.63786593 | 1.577932 | 0.040468 |
| NM_001291712 | chr14.64804268.64806268 | 1.52301 | 0.042396 |
| NM_173526 | chr14.67655109.67657109 | 1.594765 | 0.036645 |
| NM_006370 | chr14.68140602.68142602 | 1.746858 | 0.038643 |
| NM_001252650 | chr14.68161510.68163510 | 0.624444 | 0.041292 |
| NM_015351 | chr14.71107503.71109503 | 1.569408 | 0.045577 |
| NM_017791 | chr14.76043939.76045939 | 0.584504 | 0.034724 |
| NM_001267864 | chr14.78173413.78175413 | 1.922813 | 0.042047 |
| NM_001194986 | chr1.48461562.48463562 | 0.548598 | 0.048904 |
| NM_001284269 | chr14.90419901.90421901 | 1.65861 | 0.04334 |
| NM_145231 | chr14.90420121.90422121 | 1.925757 | 0.038643 |
| NM_022054 | chr14.90527108.90529108 | 0.551661 | 0.042396 |
| NM_001322227 | chr14.91525993.91527993 | 2.097477 | 0.04334 |
| NM_001284281 | chr14.91975824.91977824 | 0.437593 | 0.036645 |
| NM_001289134 | chr14.92301870.92303870 | 0.587579 | 0.04535 |
| NM_024832 | chr14.92979124.92981124 | 0.664644 | 0.046944 |
| NM_003384 | chr14.97262683.97264683 | 0.519207 | 0.041292 |
| NM_203472 | chr15.101816725.101818725 | 0.611807 | 0.04334 |
| NM_001321553 | chr15.102283972.102285972 | 1.640885 | 0.042047 |
| NM_001005326 | chr15.102344922.102346922 | 1.716017 | 0.036269 |
| NM_001349455 | chr15.25067754.25069754 | 1.967771 | 0.036645 |
| NM_001146095 | chr15.31195054.31197054 | 0.585038 | 0.041292 |
| NM_014967 | chr15.31195075.31197075 | 0.564788 | 0.036645 |
| NM_013372 | chr15.33009204.33011204 | 0.457805 | 0.034724 |
| NM_133639 | chr15.41165487.41167487 | 1.64656 | 0.04334 |
| NM_001284306 | chr15.42748765.42750765 | 1.82249 | 0.034724 |
| NM_001284307 | chr15.42782395.42784395 | 0.584055 | 0.042396 |
| NM_001321927 | chr15.43983983.43985983 | 0.632014 | 0.04535 |
| NM_001301145 | chr15.45027559.45029559 | 1.511045 | 0.045577 |
| NM_001482 | chr15.45669980.45671980 | 0.595799 | 0.034724 |
| NM_001358351 | chr15.48008563.48010563 | 1.790857 | 0.039622 |
| NM_001128610 | chr15.50715573.50717573 | 1.742463 | 0.046944 |
| NM_032802 | chr15.51056910.51058910 | 0.540179 | 0.038543 |
| NM_022841 | chr15.56534483.56536483 | 0.512614 | 0.046944 |
| NM_002755 | chr15.66678210.66680210 | 0.54387 | 0.038543 |
| NM_005902 | chr15.67357194.67359194 | 1.704208 | 0.046944 |
| NM_001190457 | chr15.68923326.68925326 | 0.591187 | 0.034724 |
| NM_001172684 | chr15.72611287.72613287 | 0.622 | 0.04535 |
| NM_005477 | chr15.73660605.73662605 | 0.503766 | 0.034724 |
| NM_015492 | chr15.75493220.75495220 | 0.591651 | 0.04334 |
| NM_006791 | chr15.79164122.79166122 | 2.077402 | 0.034724 |
| NM_205858 | chr15.85200802.85202802 | 1.516187 | 0.044041 |
| NM_001310319 | chr15.89455700.89457700 | 0.573644 | 0.040468 |
| NM_001289910 | chr15.90642853.90644853 | 1.92711 | 0.034724 |
| NM_001289823 | chr15.91413768.91415768 | 0.552987 | 0.046944 |
| NM_006011 | chr15.92936139.92938139 | 0.651885 | 0.042396 |
| NM_001288615 | chr15.99788864.99790864 | 1.502216 | 0.046944 |
| NM_001199860 | chr1.6093347.6095347 | 0.631094 | 0.046944 |
| NM_006985 | chr16.15030299.15032299 | 1.527285 | 0.036645 |
| NM_001277325 | chr16.15471151.15473151 | 1.811488 | 0.036269 |
| NM_080861 | chr16.1831581.1833581 | 0.547005 | 0.045577 |
| NM_032271 | chr16.2204798.2206798 | 0.42778 | 0.034724 |
| NM_001039 | chr16.23193039.23195039 | 0.649215 | 0.036749 |
| NM_001330520 | chr16.24739991.24741991 | 2.204394 | 0.046944 |
| NM_207013 | chr16.2826297.2828297 | 0.596087 | 0.046944 |
| NM_001014987 | chr16.28995386.28997386 | 0.623735 | 0.044041 |
| NM_001142500 | chr16.2932195.2934195 | 0.58549 | 0.04334 |
| NM_001142305 | chr16.30417734.30419734 | 1.804215 | 0.036645 |
| NM_145271 | chr16.30582728.30584728 | 0.421195 | 0.034724 |
| NM_014495 | chr1.63062157.63064157 | 1.642623 | 0.046944 |
| NM_001272096 | chr16.31043415.31045415 | 1.670492 | 0.046944 |
| NM_001311311 | chr16.31105320.31107320 | 1.520517 | 0.040468 |
| NM_024793 | chr16.3558893.3560893 | 1.510436 | 0.042047 |
| NM_012183 | chr1.63787729.63789729 | 2.132362 | 0.041292 |
| NM_133443 | chr16.46917291.46919291 | 1.593848 | 0.046944 |
| NM_148967 | chr1.6525255.6527255 | 0.665633 | 0.045577 |
| NM_001352158 | chr16.53241367.53243367 | 1.904438 | 0.046944 |
| NM_001302509 | chr16.55513444.55515444 | 0.56681 | 0.034724 |
| NM_001190158 | chr16.55988943.55990943 | 1.718227 | 0.036645 |
| NM_013410 | chr1.65612849.65614849 | 0.608059 | 0.040468 |
| NM_007006 | chr16.56484261.56486261 | 0.574982 | 0.042396 |
| NM_001324357 | chr16.56484391.56486391 | 0.635344 | 0.04334 |
| NM_018233 | chr16.56484423.56486423 | 0.634953 | 0.045577 |
| NM_018110 | chr16.57519385.57521385 | 1.675995 | 0.036645 |
| NM_024598 | chr16.58034276.58036276 | 0.527474 | 0.036749 |
| NM_001305174 | chr16.58327951.58329951 | 1.575996 | 0.038643 |
| NM_003946 | chr16.67206755.67208755 | 1.525618 | 0.038643 |
| NM_001100915 | chr16.67359674.67361674 | 1.742889 | 0.036749 |
| NM_018296 | chr16.67359746.67361746 | 1.827058 | 0.034724 |
| NM_001082487 | chr16.67693718.67695718 | 0.351246 | 0.034724 |
| NM_001037281 | chr16.67693850.67695850 | 0.339934 | 0.038643 |
| NM_001129758 | chr16.68013452.68015452 | 0.584323 | 0.035608 |
| NM_001136214 | chr16.69983607.69985607 | 0.624499 | 0.040468 |
| NM_182619 | chr16.69983804.69985804 | 0.660771 | 0.044041 |
| NM_001271197 | chr16.69983888.69985888 | 0.664745 | 0.04535 |
| NM_138383 | chr16.70718954.70720954 | 0.664317 | 0.04334 |
| NM_014761 | chr16.71928395.71930395 | 0.526008 | 0.040468 |
| NM_001142318 | chr16.72127215.72129215 | 1.682149 | 0.042047 |
| NM_001077704 | chr1.67394925.67396925 | 2.699208 | 0.036749 |
| NM_002811 | chr16.74329672.74331672 | 2.115542 | 0.034724 |
| NM_001145667 | chr16.74640042.74642042 | 0.459394 | 0.036749 |
| NM_024306 | chr16.74807729.74809729 | 0.611802 | 0.038543 |
| NM_018198 | chr1.6760966.6762966 | 0.625039 | 0.034724 |
| NM_017429 | chr16.81271295.81273295 | 1.611014 | 0.042396 |
| NM_001199742 | chr1.68149859.68151859 | 2.20897 | 0.036749 |
| NM_031463 | chr16.84177800.84179800 | 1.665036 | 0.042396 |
| NM_178452 | chr16.84177864.84179864 | 1.529571 | 0.040468 |
| NM_015144 | chr16.87524460.87526460 | 0.459899 | 0.040468 |
| NM_020655 | chr16.87635492.87637492 | 0.524108 | 0.046909 |
| NM_145109 | chr17.21186967.21188967 | 0.59469 | 0.046944 |
| NM_152464 | chr17.26683603.26685603 | 2.086516 | 0.040468 |
| NM_001290145 | chr17.26683612.26685612 | 2.026211 | 0.041292 |
| NM_001304952 | chr17.27037109.27039109 | 0.517473 | 0.046944 |
| NM_020772 | chr17.27620166.27622166 | 0.373426 | 0.044041 |
| NM_001085454 | chr17.27915610.27917610 | 1.922199 | 0.036749 |
| NM_001136498 | chr17.36885509.36887509 | 0.537214 | 0.044041 |
| NM_017748 | chr17.36980603.36982603 | 1.560689 | 0.044041 |
| NM_000978 | chr17.37009053.37011053 | 0.543463 | 0.045577 |
| NM_001838 | chr17.38720736.38722736 | 1.605802 | 0.04334 |
| NM_025233 | chr17.40713091.40715091 | 0.640155 | 0.036749 |
| NM_001320449 | chr17.4336218.4338218 | 1.575673 | 0.045577 |
| NM_014798 | chr17.43567146.43569146 | 0.397542 | 0.036269 |
| NM_006310 | chr17.45607443.45609443 | 0.472721 | 0.038643 |
| NM_001100812 | chr17.4642223.4644223 | 1.874848 | 0.042396 |
| NM_015978 | chr1.74700070.74702070 | 1.65877 | 0.041292 |
| NM_000080 | chr17.4805369.4807369 | 1.583904 | 0.04334 |
| NM_001330330 | chr17.48795904.48797904 | 1.872109 | 0.045577 |
| NM_016424 | chr17.48795925.48797925 | 1.82242 | 0.045577 |
| NM_001243877 | chr17.48944339.48946339 | 1.691502 | 0.048993 |
| NM_032530 | chr17.5094178.5096178 | 1.619384 | 0.038643 |
| NM_002126 | chr17.53341320.53343320 | 0.476992 | 0.034724 |
| NM_004645 | chr17.55037411.55039411 | 2.110099 | 0.034724 |
| NM_138962 | chr17.55332930.55334930 | 0.456119 | 0.04334 |
| NM_170721 | chr17.55333373.55335373 | 0.314166 | 0.034724 |
| NM_080677 | chr17.56159779.56161779 | 0.483432 | 0.045577 |
| NM_004687 | chr17.56594251.56596251 | 0.604978 | 0.04334 |
| NM_018488 | chr17.59532848.59534848 | 0.665128 | 0.042396 |
| NM_001174166 | chr17.66286405.66288405 | 0.488576 | 0.038643 |
| NM_001352906 | chr17.66286635.66288635 | 0.574996 | 0.046944 |
| NM_001352902 | chr17.66287211.66289211 | 0.507369 | 0.036645 |
| NM_201566 | chr17.6938393.6940393 | 0.621213 | 0.038543 |
| NM_020795 | chr17.7310501.7312501 | 1.649035 | 0.042396 |
| NM_001330499 | chr17.73850893.73852893 | 0.448959 | 0.042047 |
| NM_001348170 | chr17.73851517.73853517 | 0.408572 | 0.041292 |
| NM_033452 | chr17.73873656.73875656 | 0.469238 | 0.041292 |
| NM_001289113 | chr17.7530286.7532286 | 2.160584 | 0.04535 |
| NM_203411 | chr17.7757383.7759383 | 1.62386 | 0.04535 |
| NM_004920 | chr17.79104748.79106748 | 0.64037 | 0.036645 |
| NM_198082 | chr17.80169754.80171754 | 0.397601 | 0.035608 |
| NM_017622 | chr17.8092564.8094564 | 0.52773 | 0.046944 |
| NM_147163 | chr18.12657737.12659737 | 0.501259 | 0.035608 |
| NM_181482 | chr18.13217728.13219728 | 1.631969 | 0.04535 |
| NM_005913 | chr18.13824542.13826542 | 0.649438 | 0.041292 |
| NM_001348193 | chr18.21032184.21034184 | 1.512973 | 0.041292 |
| NM_001242508 | chr18.21890470.21892470 | 1.798314 | 0.041292 |
| NM_001160166 | chr18.22039592.22041592 | 1.50828 | 0.042396 |
| NM_001256316 | chr18.24764302.24766302 | 0.566293 | 0.042396 |
| NM_001105528 | chr18.31019045.31021045 | 1.638957 | 0.034724 |
| NM_198995 | chr18.31019685.31021685 | 1.66963 | 0.036749 |
| NM_014268 | chr18.32620323.32622323 | 1.703866 | 0.046944 |
| NM_002930 | chr18.40694657.40696657 | 1.641231 | 0.046944 |
| NM_001039535 | chr18.47900391.47902391 | 0.573642 | 0.042396 |
| NM_173629 | chr18.52257389.52259389 | 1.972321 | 0.038643 |
| NM_001348220 | chr18.53252350.53254350 | 1.760107 | 0.042396 |
| NM_001289967 | chr18.56338105.56340105 | 0.591281 | 0.036749 |
| NM_001143818 | chr18.61553938.61555938 | 1.710604 | 0.040468 |
| NM_138999 | chr18.70531934.70533934 | 1.814221 | 0.034724 |
| NM_001201465 | chr18.70533907.70535907 | 1.948587 | 0.042047 |
| NM_001025300 | chr18.8608442.8610442 | 0.592988 | 0.04334 |
| NM_019610 | chr1.89457643.89459643 | 2.07062 | 0.038643 |
| NM_207420 | chr1.9085404.9087404 | 1.543714 | 0.039622 |
| NM_012292 | chr19.1066164.1068164 | 0.508942 | 0.045577 |
| NM_001243204 | chr19.11638680.11640680 | 0.599764 | 0.045577 |
| NM_001142465 | chr19.11638987.11640987 | 0.496122 | 0.036645 |
| NM_032377 | chr19.11669051.11671051 | 0.561045 | 0.040468 |
| NM_013406 | chr19.12791701.12793701 | 0.524831 | 0.042396 |
| NM_001271043 | chr19.13134394.13136394 | 0.551263 | 0.042396 |
| NM_018467 | chr19.17325154.17327154 | 0.542073 | 0.04334 |
| NM_183242 | chr1.92544861.92546861 | 1.529265 | 0.04535 |
| NM_177543 | chr19.290169.292169 | 1.796193 | 0.045577 |
| NM_003712 | chr19.290435.292435 | 1.684097 | 0.04535 |
| NM_002067 | chr19.3093407.3095407 | 0.443912 | 0.042047 |
| NM_014727 | chr19.36207920.36209920 | 0.430056 | 0.045577 |
| NM_012237 | chr19.39389463.39391463 | 0.550243 | 0.036749 |
| NM_005884 | chr19.39615419.39617419 | 0.393272 | 0.034724 |
| NM_004596 | chr19.41255758.41257758 | 0.608984 | 0.044041 |
| NM_001256214 | chr19.42497428.42499428 | 0.514026 | 0.04535 |
| NM_015125 | chr19.42787816.42789816 | 0.363389 | 0.034724 |
| NM_173633 | chr19.42816476.42818476 | 0.644286 | 0.038643 |
| NM_001410 | chr19.42828760.42830760 | 0.523218 | 0.041292 |
| NM_006509 | chr19.45503706.45505706 | 1.730124 | 0.046909 |
| NM_145288 | chr19.45578846.45580846 | 0.604481 | 0.04334 |
| NM_001114171 | chr19.45970252.45972252 | 0.586756 | 0.039622 |
| NM_152362 | chr19.4638526.4640526 | 0.567212 | 0.04334 |
| NM_002516 | chr19.46475657.46477657 | 0.608287 | 0.045577 |
| NM_005101 | chr1.947846.949846 | 1.566499 | 0.045577 |
| NM_001331097 | chr19.48773159.48775159 | 0.559396 | 0.045577 |
| NM_018273 | chr19.48866494.48868494 | 0.564265 | 0.041292 |
| NM_012451 | chr19.48866650.48868650 | 0.597311 | 0.044041 |
| NM_013282 | chr19.4909378.4911378 | 0.464118 | 0.045577 |
| NM_001352 | chr19.49139807.49141807 | 0.426462 | 0.036749 |
| NM_016553 | chr19.50431988.50433988 | 1.786616 | 0.040468 |
| NM_032712 | chr19.51306811.51308811 | 1.8235 | 0.042396 |
| NM_003830 | chr19.52132727.52134727 | 0.422419 | 0.041292 |
| NM_001172654 | chr19.54803221.54805221 | 1.603783 | 0.04535 |
| NM_021216 | chr19.57105663.57107663 | 1.988192 | 0.044041 |
| NM_001146186 | chr19.57335374.57337374 | 1.641986 | 0.038643 |
| NM_017908 | chr19.58986530.58988530 | 0.574095 | 0.046944 |
| NM_001316982 | chr19.59049278.59051278 | 0.567972 | 0.041292 |
| NM_001220500 | chr19.7766032.7768032 | 0.569928 | 0.036645 |
| NM_020248 | chr1.9969316.9971316 | 0.521707 | 0.039622 |
| NM_001136566 | chr20.1205763.1207763 | 0.446666 | 0.044041 |
| NM_001163023 | chr20.21105615.21107615 | 0.616549 | 0.04535 |
| NM_080610 | chr20.23548386.23550386 | 1.886081 | 0.034724 |
| NM_002862 | chr20.25227705.25229705 | 0.56862 | 0.045577 |
| NM_001037731 | chr20.29895388.29897388 | 1.538015 | 0.04535 |
| NM_153323 | chr20.29977452.29979452 | 1.517117 | 0.039622 |
| NM_030811 | chr20.3025674.3027674 | 0.460939 | 0.032921 |
| NM_177991 | chr20.30457044.30459044 | 0.598021 | 0.045577 |
| NM_182658 | chr20.31642136.31644136 | 1.525024 | 0.034724 |
| NM_001324238 | chr20.3188513.3190513 | 1.843126 | 0.034724 |
| NM_001324240 | chr20.3189005.3191005 | 1.566474 | 0.045577 |
| NM_003098 | chr20.32030698.32032698 | 0.50192 | 0.044041 |
| NM_001174089 | chr20.3218887.3220887 | 0.528568 | 0.038543 |
| NM_001323681 | chr20.326369.328369 | 0.417849 | 0.034724 |
| NM_001281729 | chr20.33103188.33105188 | 0.581969 | 0.036645 |
| NM_001329429 | chr20.33291111.33293111 | 0.648723 | 0.038643 |
| NM_001355008 | chr20.33864981.33866981 | 1.78295 | 0.042396 |
| NM_080834 | chr20.34555502.34557502 | 1.600453 | 0.034724 |
| NM_145762 | chr20.3643046.3645046 | 1.634217 | 0.038643 |
| NM_080607 | chr20.36530498.36532498 | 0.579169 | 0.034724 |
| NM_001324193 | chr20.3869448.3871448 | 1.601943 | 0.046944 |
| NM_001302861 | chr20.44175065.44177065 | 1.748742 | 0.034724 |
| NM_001348187 | chr20.47834831.47836831 | 1.932359 | 0.044041 |
| NM_001319135 | chr20.47834832.47836832 | 1.932359 | 0.044041 |
| NM_015266 | chr20.48428249.48430249 | 1.978009 | 0.036645 |
| NM_002623 | chr20.52823501.52825501 | 0.594929 | 0.039622 |
| NM_080617 | chr20.54579528.54581528 | 0.634854 | 0.046944 |
| NM_012444 | chr20.55903830.55905830 | 1.619823 | 0.036269 |
| NM_006242 | chr20.58514352.58516352 | 0.603585 | 0.042396 |
| NM_001190827 | chr20.58514443.58516443 | 0.605604 | 0.044041 |
| NM_001819 | chr20.5890973.5892973 | 1.600138 | 0.045577 |
| NM_033405 | chr20.62198107.62200107 | 0.533137 | 0.036269 |
| NM_012469 | chr20.62611430.62613430 | 1.64825 | 0.046944 |
| NM_005873 | chr20.62709845.62711845 | 0.566178 | 0.04334 |
| NM_173647 | chr2.101924178.101926178 | 2.686873 | 0.039622 |
| NM_001318896 | chr2.106014575.106016575 | 0.57571 | 0.042396 |
| NM_001253876 | chr2.106754337.106756337 | 0.663409 | 0.04334 |
| NM_006267 | chr2.109334936.109336936 | 0.55786 | 0.042047 |
| NM_001278616 | chr2.111434684.111436684 | 0.450822 | 0.036645 |
| NM_182500 | chr2.11272178.11274178 | 1.67505 | 0.042396 |
| NM_001304361 | chr2.113521254.113523254 | 1.587396 | 0.040468 |
| NM_001349205 | chr2.11887658.11889658 | 0.588247 | 0.034724 |
| NM_002881 | chr2.121009413.121011413 | 0.546073 | 0.044041 |
| NM_001352591 | chr21.22369613.22371613 | 0.613997 | 0.036269 |
| NM_001197297 | chr21.27106257.27108257 | 0.513212 | 0.04334 |
| NM_001161416 | chr2.128402438.128404438 | 0.642119 | 0.038643 |
| NM_001286619 | chr21.30463574.30465574 | 1.570476 | 0.046944 |
| NM_001171083 | chr2.130938330.130940330 | 0.602502 | 0.04334 |
| NM_181600 | chr21.31801571.31803571 | 1.625626 | 0.038643 |
| NM_080386 | chr2.132232579.132234579 | 0.598625 | 0.040468 |
| NM_001289128 | chr21.34601199.34603199 | 0.492308 | 0.042396 |
| NM_001199241 | chr2.143634194.143636194 | 1.743088 | 0.04535 |
| NM_016818 | chr21.43638266.43640266 | 1.540126 | 0.046944 |
| NM_207174 | chr21.43639007.43641007 | 1.839223 | 0.038543 |
| NM_003681 | chr21.45137977.45139977 | 0.444236 | 0.039622 |
| NM_002626 | chr21.45718916.45720916 | 0.365063 | 0.034724 |
| NM_198687 | chr21.45992605.45994605 | 0.571507 | 0.036749 |
| NM_001350598 | chr21.47570288.47572288 | 0.638055 | 0.038643 |
| NM_007115 | chr2.152213105.152215105 | 1.836243 | 0.034724 |
| NM_006186 | chr2.157188287.157190287 | 0.594258 | 0.038643 |
| NM_001009959 | chr2.158183225.158185225 | 1.561769 | 0.046944 |
| NM_001282390 | chr2.161055824.161057824 | 2.123836 | 0.034724 |
| NM_002897 | chr2.161349318.161351318 | 0.648942 | 0.042047 |
| NM_001199135 | chr2.162015938.162017938 | 1.93667 | 0.042396 |
| NM_005805 | chr2.162163785.162165785 | 1.658918 | 0.045577 |
| NM_018086 | chr2.164591517.164593517 | 0.632514 | 0.046944 |
| NM_021176 | chr2.169756749.169758749 | 1.813098 | 0.034724 |
| NM_001083615 | chr2.171033654.171035654 | 1.764582 | 0.036645 |
| NM_001256909 | chr2.172377756.172379756 | 0.616989 | 0.035608 |
| NM_024843 | chr2.172377865.172379865 | 0.639041 | 0.034724 |
| NM_001271785 | chr2.172543181.172545181 | 1.527085 | 0.04535 |
| NM_003642 | chr2.172777934.172779934 | 0.643333 | 0.044041 |
| NM_001316306 | chr2.173291081.173293081 | 0.635045 | 0.04334 |
| NM_001079818 | chr2.173291282.173293282 | 0.643674 | 0.04535 |
| NM_001321233 | chr2.17710681.17712681 | 2.311046 | 0.034724 |
| NM_001077358 | chr2.178786564.178788564 | 1.52637 | 0.045577 |
| NM_016953 | chr2.178936482.178938482 | 1.583974 | 0.038643 |
| NM_001130009 | chr2.17934426.17936426 | 0.585278 | 0.041292 |
| NM_001271581 | chr2.183579767.183581767 | 0.521797 | 0.036749 |
| NM_000090 | chr2.189838098.189840098 | 1.519694 | 0.044041 |
| NM_020760 | chr2.197456408.197458408 | 0.385629 | 0.034724 |
| NM_012086 | chr2.197663492.197665492 | 0.558955 | 0.04334 |
| NM_001321694 | chr2.201675624.201677624 | 0.567886 | 0.045577 |
| NM_001321691 | chr2.201675644.201677644 | 0.53838 | 0.045577 |
| NM_001202515 | chr2.202003997.202005997 | 1.954426 | 0.036645 |
| NM_139163 | chr2.202221121.202223121 | 1.558551 | 0.039622 |
| NM_001282791 | chr2.20526147.20528147 | 1.719873 | 0.038643 |
| NM_015040 | chr2.209129990.209131990 | 1.508886 | 0.046944 |
| NM_001039538 | chr2.210287770.210289770 | 1.840108 | 0.044041 |
| NM_015657 | chr2.215895810.215897810 | 1.590489 | 0.034724 |
| NM_212478 | chr2.216299791.216301791 | 0.612979 | 0.045577 |
| NM_001306130 | chr2.216299793.216301793 | 0.612979 | 0.045577 |
| NM_017829 | chr22.17645177.17647177 | 1.774592 | 0.039622 |
| NM_000407 | chr22.19710065.19712065 | 0.583595 | 0.036749 |
| NM_025216 | chr2.219744254.219746254 | 1.538556 | 0.036269 |
| NM_003936 | chr2.219823349.219825349 | 0.632765 | 0.045577 |
| NM_024627 | chr22.19841371.19843371 | 0.570335 | 0.036645 |
| NM_053004 | chr22.19841462.19843462 | 0.545622 | 0.036269 |
| NM_001349828 | chr2.220082739.220084739 | 0.602203 | 0.041292 |
| NM_001355221 | chr2.220116964.220118964 | 1.768848 | 0.042047 |
| NM_001243537 | chr22.20136431.20138431 | 0.653742 | 0.038643 |
| NM_001128633 | chr22.21736662.21738662 | 2.072021 | 0.038643 |
| NM_001128635 | chr22.21737039.21739039 | 1.742635 | 0.044041 |
| NM_001318127 | chr22.22895603.22897603 | 1.596308 | 0.040468 |
| NM_153038 | chr2.223161865.223163865 | 0.645111 | 0.04334 |
| NM_003469 | chr2.224466217.224468217 | 1.722646 | 0.034724 |
| NM_016327 | chr22.24890250.24892250 | 0.636317 | 0.036269 |
| NM_001288833 | chr22.24998123.25000123 | 1.599398 | 0.038643 |
| NM_001202502 | chr22.29976085.29978085 | 0.58254 | 0.034724 |
| NM_001353443 | chr22.30937302.30939302 | 1.512305 | 0.046944 |
| NM_001282738 | chr22.31088768.31090768 | 1.545932 | 0.044041 |
| NM_001303256 | chr22.31363273.31365273 | 1.769228 | 0.046944 |
| NM_015372 | chr22.32340348.32342348 | 0.543013 | 0.036269 |
| NM_018410 | chr2.234762212.234764212 | 0.626676 | 0.04535 |
| NM_014521 | chr2.235859627.235861627 | 0.503325 | 0.034724 |
| NM_001166343 | chr22.37881425.37883425 | 1.72067 | 0.046944 |
| NM_001195071 | chr22.38667670.38669670 | 0.51482 | 0.039622 |
| NM_001199580 | chr22.39150467.39152467 | 0.578416 | 0.036749 |
| NM_016272 | chr22.41842027.41844027 | 0.553036 | 0.042047 |
| NM_001271978 | chr2.242446775.242448775 | 0.532795 | 0.040468 |
| NM_001282305 | chr2.242447987.242449987 | 0.606822 | 0.038643 |
| NM_001164356 | chr2.242555916.242557916 | 0.555663 | 0.036269 |
| NM_001206802 | chr2.24306201.24308201 | 0.568136 | 0.036269 |
| NM_022785 | chr22.44207217.44209217 | 0.665034 | 0.036749 |
| NM_013236 | chr22.46066677.46068677 | 0.550623 | 0.041292 |
| NM_001256308 | chr2.25872091.25874091 | 0.604699 | 0.038643 |
| NM_080592 | chr2.27433898.27435898 | 0.608783 | 0.042047 |
| NM_001142634 | chr2.29032699.29034699 | 1.873499 | 0.040468 |
| NM_005102 | chr2.36824332.36826332 | 0.584784 | 0.04334 |
| NM_001170792 | chr2.38154901.38156901 | 1.631671 | 0.046944 |
| NM_001145451 | chr2.39145503.39147503 | 1.588801 | 0.045577 |
| NM_001282756 | chr2.42794184.42796184 | 1.848581 | 0.038543 |
| NM_005413 | chr2.45168036.45170036 | 0.610786 | 0.046944 |
| NM_001005369 | chr2.55495315.55497315 | 0.577066 | 0.039622 |
| NM_001321004 | chr2.55495384.55497384 | 0.569892 | 0.039622 |
| NM_001348514 | chr2.56410257.56412257 | 0.609691 | 0.045577 |
| NM_203437 | chr2.64750438.64752438 | 1.741463 | 0.038643 |
| NM_001329917 | chr2.68383961.68385961 | 1.681299 | 0.04535 |
| NM_006857 | chr2.70120074.70122074 | 1.831249 | 0.042047 |
| NM_001134462 | chr2.73428385.73430385 | 1.519219 | 0.04334 |
| NM_001330386 | chr2.73963533.73965533 | 0.49784 | 0.04535 |
| NM_001353344 | chr2.74034865.74036865 | 1.567826 | 0.040468 |
| NM_006636 | chr2.74424689.74426689 | 0.611226 | 0.046944 |
| NM_032779 | chr2.74709357.74711357 | 1.721362 | 0.042047 |
| NM_133637 | chr2.74752408.74754408 | 0.498702 | 0.034724 |
| NM_005911 | chr2.85765100.85767100 | 1.877544 | 0.041292 |
| NM_006590 | chr2.85842214.85844214 | 0.531659 | 0.038643 |
| NM_001354234 | chr2.86114870.86116870 | 1.566356 | 0.045577 |
| NM_001100170 | chr2.86421893.86423893 | 2.034218 | 0.038643 |
| NM_002665 | chr2.88046605.88048605 | 1.590786 | 0.034724 |
| NM_001032392 | chr2.88046609.88048609 | 1.590786 | 0.034724 |
| NM_001330364 | chr2.88366298.88368298 | 1.586391 | 0.034724 |
| NM_000682 | chr2.96780984.96782984 | 0.562159 | 0.045577 |
| NM_001142292 | chr2.97404813.97406813 | 1.879056 | 0.036645 |
| NM_001322351 | chr2.97404829.97406829 | 1.953715 | 0.036645 |
| NM_001320419 | chr2.99796524.99798524 | 1.846203 | 0.045577 |
| NM_005783 | chr2.99951860.99953860 | 1.615853 | 0.048904 |
| NM_016247 | chr3.101038419.101040419 | 1.544263 | 0.04535 |
| NM_001282801 | chr3.101231085.101233085 | 1.546904 | 0.045577 |
| NM_001353564 | chr3.10660646.10662646 | 1.659443 | 0.034724 |
| NM_001348906 | chr3.112279166.112281166 | 1.756133 | 0.045577 |
| NM_001348905 | chr3.112279526.112281526 | 1.641443 | 0.046944 |
| NM_001164343 | chr3.114789261.114791261 | 2.17058 | 0.034724 |
| NM_002338 | chr3.116163385.116165385 | 0.646479 | 0.045577 |
| NM_001353326 | chr3.118752813.118754813 | 2.011714 | 0.036749 |
| NM_152305 | chr3.119186784.119188784 | 1.592466 | 0.038643 |
| NM_016589 | chr3.119216367.119218367 | 1.712966 | 0.038643 |
| NM_024610 | chr3.122511666.122513666 | 1.659101 | 0.036749 |
| NM_001320728 | chr3.122511674.122513674 | 1.659101 | 0.036749 |
| NM_005037 | chr3.12328348.12330348 | 0.660163 | 0.042396 |
| NM_001317775 | chr3.123710017.123712017 | 0.655124 | 0.042047 |
| NM_001348432 | chr3.125092553.125094553 | 1.58886 | 0.042396 |
| NM_002950 | chr3.128368719.128370719 | 0.481036 | 0.046944 |
| NM_001330636 | chr3.13520664.13522664 | 0.587031 | 0.04334 |
| NM_024827 | chr3.13520714.13522714 | 0.581777 | 0.044041 |
| NM_001136041 | chr3.13520843.13522843 | 0.519239 | 0.035608 |
| NM_001190796 | chr3.136648316.136650316 | 1.78698 | 0.034724 |
| NM_001200047 | chr3.139395892.139397892 | 0.543149 | 0.040468 |
| NM_052995 | chr3.150661023.150663023 | 1.632431 | 0.034724 |
| NM_001256819 | chr3.150689786.150691786 | 1.628293 | 0.038643 |
| NM_004844 | chr3.15373136.15375136 | 0.559399 | 0.036645 |
| NM_001038705 | chr3.154146504.154148504 | 1.56011 | 0.041292 |
| NM_001308166 | chr3.158361316.158363316 | 1.78206 | 0.04535 |
| NM_025047 | chr3.160393947.160395947 | 1.83248 | 0.036645 |
| NM_001317912 | chr3.160559000.160561000 | 1.773926 | 0.039622 |
| NM_001135095 | chr3.171757343.171759343 | 0.527567 | 0.044041 |
| NM_001349101 | chr3.172467741.172469741 | 1.561708 | 0.04535 |
| NM_014932 | chr3.173115237.173117237 | 1.5935 | 0.038643 |
| NM_001303425 | chr3.179039778.179041778 | 0.594259 | 0.038643 |
| NM_005087 | chr3.180629233.180631233 | 0.591999 | 0.045577 |
| NM_138345 | chr3.183947216.183949216 | 0.483026 | 0.042047 |
| NM_032331 | chr3.183966444.183968444 | 0.455776 | 0.039622 |
| NM_024524 | chr3.194187968.194189968 | 1.682199 | 0.044041 |
| NM_001145642 | chr3.197475570.197477570 | 1.52898 | 0.04334 |
| NM_001199257 | chr3.20226725.20228725 | 0.589498 | 0.034724 |
| NM_018297 | chr3.25823989.25825989 | 1.537621 | 0.044041 |
| NM_001317809 | chr3.26664571.26666571 | 1.505871 | 0.044041 |
| NM_001304384 | chr3.27257014.27259014 | 2.405071 | 0.032921 |
| NM_001177712 | chr3.29321802.29323802 | 1.756665 | 0.040468 |
| NM_000564 | chr3.3151058.3153058 | 1.619904 | 0.042047 |
| NM_178862 | chr3.31572992.31574992 | 0.41743 | 0.036749 |
| NM_001130410 | chr3.38177733.38179733 | 0.513423 | 0.042396 |
| NM_001296 | chr3.42849963.42851963 | 1.934555 | 0.034724 |
| NM_182760 | chr3.4507966.4509966 | 1.620346 | 0.038643 |
| NM_001271022 | chr3.48487113.48489113 | 0.511407 | 0.044041 |
| NM_177939 | chr3.49026340.49028340 | 1.634432 | 0.036645 |
| NM_199070 | chr3.49058073.49060073 | 0.646483 | 0.04535 |
| NM_001320581 | chr3.49130065.49132065 | 1.657106 | 0.045577 |
| NM_001080528 | chr3.49228291.49230291 | 1.505383 | 0.034724 |
| NM_003670 | chr3.5020096.5022096 | 1.623953 | 0.04535 |
| NM_001290060 | chr3.50305426.50307426 | 0.600341 | 0.042396 |
| NM_001290061 | chr3.50305592.50307592 | 0.591953 | 0.040468 |
| NM_203424 | chr3.51894644.51896644 | 1.748608 | 0.035608 |
| NM_007284 | chr3.52272183.52274183 | 2.548334 | 0.034724 |
| NM_001353151 | chr3.56590183.56592183 | 2.426222 | 0.035608 |
| NM_012096 | chr3.57260764.57262764 | 1.604279 | 0.042396 |
| NM_001311178 | chr3.57874640.57876640 | 1.798661 | 0.041292 |
| NM_001349498 | chr3.58318032.58320032 | 1.958945 | 0.038643 |
| NM_001282713 | chr3.58571840.58573840 | 0.586553 | 0.038643 |
| NM_001278689 | chr3.69062045.69064045 | 0.657381 | 0.045577 |
| NM_020872 | chr3.74569291.74571291 | 2.28997 | 0.034724 |
| NM_001005338 | chr3.97850541.97852541 | 1.605686 | 0.04535 |
| NM_001004737 | chr3.98215524.98217524 | 1.502257 | 0.042047 |
| NM_001321144 | chr3.9920938.9922938 | 1.675331 | 0.038543 |
| NM_000671 | chr4.100008939.100010939 | 1.925806 | 0.038643 |
| NM_001278311 | chr4.100866883.100868883 | 1.564974 | 0.038643 |
| NM_001318085 | chr4.110353870.110355870 | 1.552095 | 0.046944 |
| NM_198506 | chr4.110768339.110770339 | 1.686165 | 0.038643 |
| NM_015454 | chr4.113557611.113559611 | 1.555009 | 0.042396 |
| NM_001354269 | chr4.113626173.113628173 | 0.65317 | 0.04334 |
| NM_001354245 | chr4.113969569.113971569 | 1.622806 | 0.039622 |
| NM_033437 | chr4.120548239.120550239 | 0.574891 | 0.044041 |
| NM_003305 | chr4.122853268.122855268 | 0.635673 | 0.036645 |
| NM_012118 | chr4.139935912.139937912 | 0.574359 | 0.042396 |
| NM_001277353 | chr4.141418531.141420531 | 1.641032 | 0.034724 |
| NM_017493 | chr4.146094896.146096896 | 1.957623 | 0.038643 |
| NM_004564 | chr4.152681175.152683175 | 1.742795 | 0.039622 |
| NM_021871 | chr4.155510918.155512918 | 0.582327 | 0.036749 |
| NM_005130 | chr4.15939363.15941363 | 1.72386 | 0.034724 |
| NM_201591 | chr4.176733265.176735265 | 1.992308 | 0.034724 |
| NM_170710 | chr4.176985984.176987984 | 1.725078 | 0.034724 |
| NM_001351747 | chr4.183837406.183839406 | 0.523218 | 0.041292 |
| NM_001351753 | chr4.183837630.183839630 | 0.63928 | 0.046944 |
| NM_001286711 | chr4.185746268.185748268 | 0.490217 | 0.039622 |
| NM_145290 | chr4.22516677.22518677 | 0.565123 | 0.036269 |
| NM_001358 | chr4.24585184.24587184 | 2.81032 | 0.032921 |
| NM_001185010 | chr4.2469794.2471794 | 0.465791 | 0.044041 |
| NM_001292054 | chr4.26584545.26586545 | 1.551655 | 0.042396 |
| NM_001286645 | chr4.2844876.2846876 | 0.593141 | 0.04334 |
| NM_138389 | chr4.38868353.38870353 | 0.554686 | 0.042047 |
| NM_001184701 | chr4.39528218.39530218 | 1.703536 | 0.042047 |
| NM_001287764 | chr4.4386982.4388982 | 0.524242 | 0.042396 |
| NM_014392 | chr4.4387689.4389689 | 0.39494 | 0.034724 |
| NM_152540 | chr4.54231242.54233242 | 0.518926 | 0.034724 |
| NM_032313 | chr4.57842826.57844826 | 2.687368 | 0.034724 |
| NM_001010874 | chr4.65274178.65276178 | 2.123694 | 0.034724 |
| NM_012108 | chr4.68423414.68425414 | 1.839954 | 0.042396 |
| NM_001290091 | chr4.69885115.69887115 | 1.855984 | 0.042047 |
| NM_000200 | chr4.70893129.70895129 | 1.537715 | 0.034724 |
| NM_001354717 | chr4.74300935.74302935 | 2.049181 | 0.034724 |
| NM_002416 | chr4.76927676.76929676 | 1.533469 | 0.039622 |
| NM_001042784 | chr4.77327458.77329458 | 2.119399 | 0.036645 |
| NM_001029870 | chr4.77818079.77820079 | 0.44628 | 0.038643 |
| NM_203423 | chr4.7939727.7941727 | 0.557772 | 0.038543 |
| NM_001286781 | chr4.80993477.80995477 | 1.740621 | 0.049794 |
| NM_001286780 | chr4.80993626.80995626 | 1.647791 | 0.044041 |
| NM_001291812 | chr4.81188418.81190418 | 0.593327 | 0.034724 |
| NM_001199830 | chr4.84254966.84256966 | 0.611752 | 0.045577 |
| NM_031305 | chr4.86850425.86852425 | 1.59955 | 0.036645 |
| NM_001184696 | chr4.88753120.88755120 | 2.378338 | 0.034724 |
| NM_173488 | chr5.101833720.101835720 | 0.647762 | 0.04535 |
| NM_001308028 | chr5.108083627.108085627 | 1.947014 | 0.044041 |
| NM_001308031 | chr5.108259306.108261306 | 1.603477 | 0.042047 |
| NM_001354899 | chr5.112072555.112074555 | 1.666117 | 0.042047 |
| NM_001242377 | chr5.112311406.112313406 | 0.513223 | 0.038643 |
| NM_000758 | chr5.131408484.131410484 | 1.834968 | 0.034724 |
| NM_001745 | chr5.134073169.134075169 | 0.657192 | 0.046944 |
| NM_020957 | chr5.140560264.140562264 | 1.663725 | 0.04334 |
| NM_022481 | chr5.141060800.141062800 | 0.558759 | 0.036645 |
| NM_030571 | chr5.141487323.141489323 | 0.580912 | 0.039622 |
| NM_030964 | chr5.141703620.141705620 | 0.554691 | 0.040468 |
| NM_001354956 | chr5.142064268.142066268 | 1.762439 | 0.038543 |
| NM_021182 | chr5.143190725.143192725 | 1.601586 | 0.040468 |
| NM_001024947 | chr5.143549278.143551278 | 0.625866 | 0.044041 |
| NM_020768 | chr5.143549436.143551436 | 0.604063 | 0.036749 |
| NM_001044 | chr5.1444543.1446543 | 0.619823 | 0.04535 |
| NM_003122 | chr5.147217794.147219794 | 1.618208 | 0.04334 |
| NM_052860 | chr5.150283545.150285545 | 1.685304 | 0.045577 |
| NM_001301083 | chr5.154236808.154238808 | 1.800302 | 0.038643 |
| NM_001301082 | chr5.154237076.154239076 | 1.500877 | 0.04535 |
| NM_001301077 | chr5.154237078.154239078 | 1.500877 | 0.04535 |
| NM_001252156 | chr5.154316776.154318776 | 0.611802 | 0.042047 |
| NM_005546 | chr5.156606906.156608906 | 1.610681 | 0.039622 |
| NM_016093 | chr5.172385426.172387426 | 0.60745 | 0.042047 |
| NM_001205 | chr5.172570444.172572444 | 1.714201 | 0.034724 |
| NM_001099408 | chr5.176056682.176058682 | 1.638475 | 0.045577 |
| NM_198868 | chr5.179333856.179335856 | 0.587539 | 0.044041 |
| NM_001317227 | chr5.22211878.22213878 | 1.540976 | 0.046944 |
| NM_016279 | chr5.27037689.27039689 | 1.562205 | 0.038643 |
| NM_001145522 | chr5.34655432.34657432 | 1.78471 | 0.04535 |
| NM_001145520 | chr5.34655595.34657595 | 1.810638 | 0.04535 |
| NM_001145523 | chr5.34686663.34688663 | 1.739545 | 0.036269 |
| NM_001349333 | chr5.39218708.39220708 | 1.601295 | 0.034724 |
| NM_001258287 | chr5.54987881.54989881 | 1.729598 | 0.044041 |
| NM_001349243 | chr5.59782925.59784925 | 1.730921 | 0.036749 |
| NM_001164664 | chr5.65891175.65893175 | 0.604387 | 0.044041 |
| NM_133341 | chr5.68665861.68667861 | 1.701316 | 0.04535 |
| NM_001297716 | chr5.75378238.75380238 | 0.600523 | 0.038543 |
| NM_032567 | chr5.79614789.79616789 | 1.521198 | 0.034724 |
| NM_147147 | chr6.105583221.105585221 | 2.22291 | 0.032921 |
| NM_014028 | chr6.108394941.108396941 | 0.546086 | 0.034724 |
| NM_022765 | chr6.109776190.109778190 | 0.657753 | 0.04334 |
| NM_207582 | chr6.11111071.11113071 | 1.678107 | 0.036749 |
| NM_001164283 | chr6.111887563.111889563 | 1.657542 | 0.042047 |
| NM_173674 | chr6.117802766.117804766 | 1.591644 | 0.036645 |
| NM_001168319 | chr6.12289528.12291528 | 1.65015 | 0.038643 |
| NM_001318907 | chr6.125523784.125525784 | 1.775248 | 0.040468 |
| NM_014702 | chr6.127779535.127781535 | 1.573231 | 0.045577 |
| NM_001010905 | chr6.127897318.127899318 | 1.775432 | 0.035608 |
| NM_001258277 | chr6.130686425.130688425 | 0.611945 | 0.044041 |
| NM_033260 | chr6.1311674.1313674 | 0.530076 | 0.042047 |
| NM_001252660 | chr6.131320907.131322907 | 1.609146 | 0.036645 |
| NM_138633 | chr6.131570298.131572298 | 1.606082 | 0.046944 |
| NM_001244438 | chr6.131893343.131895343 | 1.744637 | 0.038643 |
| NM_004830 | chr6.131948379.131950379 | 1.790925 | 0.04334 |
| NM_000288 | chr6.137142701.137144701 | 1.660794 | 0.039622 |
| NM_001195037 | chr6.139116247.139118247 | 1.516484 | 0.039622 |
| NM_006079 | chr6.139694787.139696787 | 0.599098 | 0.045577 |
| NM_001358410 | chr6.142453295.142455295 | 1.892968 | 0.034724 |
| NM_015718 | chr6.155776037.155778037 | 1.502394 | 0.042396 |
| NM_001303253 | chr6.160182510.160184510 | 2.023822 | 0.042396 |
| NM_001322817 | chr6.160182561.160184561 | 2.054693 | 0.042047 |
| NM_001129895 | chr6.168376619.168378619 | 0.596986 | 0.046944 |
| NM_005618 | chr6.170598697.170600697 | 0.654744 | 0.049794 |
| NM_001286379 | chr6.170598790.170600790 | 0.653825 | 0.049794 |
| NM_006355 | chr6.25961916.25963916 | 1.647933 | 0.042396 |
| NM_138720 | chr6.26157348.26159348 | 0.630009 | 0.046944 |
| NM_001197249 | chr6.26364386.26366386 | 1.720677 | 0.035608 |
| NM_001347872 | chr6.2769564.2771564 | 1.570851 | 0.036645 |
| NM_003536 | chr6.27776841.27778841 | 0.61122 | 0.046944 |
| NM_001005226 | chr6.29054090.29056090 | 1.660235 | 0.040468 |
| NM_030905 | chr6.29140310.29142310 | 1.939848 | 0.036749 |
| NM_007160 | chr6.29554682.29556682 | 1.689014 | 0.034724 |
| NM_001242758 | chr6.29909246.29911246 | 0.526999 | 0.040468 |
| NM_001243042 | chr6.31238913.31240913 | 2.074502 | 0.04535 |
| NM_001177519 | chr6.31370343.31372343 | 0.654064 | 0.044041 |
| NM_148919 | chr6.32810816.32812816 | 0.513367 | 0.044041 |
| NM_033554 | chr6.33040454.33042454 | 1.709832 | 0.044041 |
| NM_015482 | chr6.3455793.3457793 | 0.646922 | 0.042396 |
| NM_207409 | chr6.35743370.35745370 | 0.529387 | 0.038643 |
| NM_183373 | chr6.3751246.3753246 | 0.415401 | 0.042047 |
| NM_001359094 | chr6.40345075.40347075 | 1.51887 | 0.041292 |
| NM_001159726 | chr6.41009456.41011456 | 0.529043 | 0.034724 |
| NM_006653 | chr6.41746643.41748643 | 0.5148 | 0.046944 |
| NM_001287427 | chr6.41908586.41910586 | 0.403454 | 0.045577 |
| NM_002098 | chr6.42161694.42163694 | 1.703857 | 0.041292 |
| NM_006443 | chr6.43196211.43198211 | 0.531693 | 0.042396 |
| NM_001253 | chr6.44354250.44356250 | 0.627764 | 0.044041 |
| NM_005588 | chr6.46760093.46762093 | 1.962873 | 0.034724 |
| NM_181744 | chr6.47748774.47750774 | 1.975231 | 0.034724 |
| NM_001010872 | chr6.54710568.54712568 | 1.531164 | 0.034724 |
| NM_001318754 | chr6.55955459.55957459 | 1.544769 | 0.04334 |
| NM_018368 | chr6.70506049.70508049 | 1.819457 | 0.036269 |
| NM_001281439 | chr6.71376473.71378473 | 2.065008 | 0.034724 |
| NM_001159588 | chr6.74404807.74406807 | 2.34555 | 0.036749 |
| NM_001100409 | chr6.76310224.76312224 | 1.759931 | 0.038643 |
| NM_001563 | chr6.76781395.76783395 | 1.632012 | 0.04535 |
| NM_002395 | chr6.84139938.84141938 | 0.550792 | 0.041292 |
| NM_002526 | chr6.86158301.86160301 | 1.657395 | 0.040468 |
| NM_001168398 | chr6.88181642.88183642 | 2.675123 | 0.034724 |
| NM_006813 | chr6.89789428.89791428 | 2.212408 | 0.039622 |
| NM_003188 | chr6.91296020.91298020 | 0.456903 | 0.034724 |
| NM_003227 | chr7.100238173.100240173 | 0.593958 | 0.034724 |
| NM_001302621 | chr7.100492592.100494592 | 0.491307 | 0.04334 |
| NM_001302622 | chr7.100492754.100494754 | 0.50083 | 0.044041 |
| NM_001350738 | chr7.105028377.105030377 | 1.734711 | 0.039622 |
| NM_001350740 | chr7.105028837.105030837 | 2.106015 | 0.034724 |
| NM_001282427 | chr7.106504722.106506722 | 1.913707 | 0.042047 |
| NM_002649 | chr7.106504923.106506923 | 2.099864 | 0.036749 |
| NM_005295 | chr7.107109501.107111501 | 1.744432 | 0.041292 |
| NM_138445 | chr7.1093910.1095910 | 0.604975 | 0.036645 |
| NM_002489 | chr7.10978813.10980813 | 1.704526 | 0.039622 |
| NM_015641 | chr7.115849546.115851546 | 0.652499 | 0.042396 |
| NM_001324402 | chr7.116311412.116313412 | 0.556098 | 0.036645 |
| NM_001127500 | chr7.116311443.116313443 | 0.546954 | 0.036645 |
| NM_001201372 | chr7.128430463.128432463 | 0.554648 | 0.034724 |
| NM_016478 | chr7.129690291.129692291 | 1.729184 | 0.038643 |
| NM_014997 | chr7.129709348.129711348 | 1.894812 | 0.034724 |
| NM_138693 | chr7.130417860.130419860 | 0.453829 | 0.036749 |
| NM_033140 | chr7.134575150.134577150 | 1.509179 | 0.036645 |
| NM_001321386 | chr7.136952602.136954602 | 1.532793 | 0.042047 |
| NM_001321709 | chr7.137530078.137532078 | 0.581332 | 0.04535 |
| NM_173569 | chr7.138915230.138917230 | 0.577632 | 0.038643 |
| NM_001270643 | chr7.139043591.139045591 | 1.673783 | 0.038643 |
| NM_001080511 | chr7.139207673.139209673 | 1.823317 | 0.041292 |
| NM_013446 | chr7.140178369.140180369 | 0.585592 | 0.042396 |
| NM_019841 | chr7.142629820.142631820 | 1.579767 | 0.038643 |
| NM_001224 | chr7.142985680.142987680 | 1.563523 | 0.046944 |
| NM_001206941 | chr7.143581466.143583466 | 0.516473 | 0.034724 |
| NM_001080413 | chr7.144106320.144108320 | 1.605993 | 0.042047 |
| NM_001289990 | chr7.149569056.149571056 | 1.795414 | 0.04535 |
| NM_013400 | chr7.150064878.150066878 | 0.430903 | 0.036749 |
| NM_003040 | chr7.150755656.150757656 | 0.540673 | 0.045577 |
| NM_032581 | chr7.23052770.23054770 | 2.411339 | 0.036749 |
| NM_004403 | chr7.24796639.24798639 | 1.654947 | 0.041292 |
| NM_000522 | chr7.27238725.27240725 | 0.55171 | 0.044041 |
| NM_182898 | chr7.28451143.28453143 | 1.591101 | 0.045577 |
| NM_001293072 | chr7.29236356.29238356 | 1.614802 | 0.038643 |
| NM_001293080 | chr7.29518327.29520327 | 1.617195 | 0.039622 |
| NM_001080529 | chr7.29845169.29847169 | 1.597373 | 0.035608 |
| NM_001324281 | chr7.3082579.3084579 | 0.56696 | 0.034724 |
| NM_001257968 | chr7.31568075.31570075 | 1.738728 | 0.039622 |
| NM_001130710 | chr7.32533870.32535870 | 2.077696 | 0.035608 |
| NM_015060 | chr7.32534037.32536037 | 2.42085 | 0.034724 |
| NM_032016 | chr7.38216807.38218807 | 1.579597 | 0.04334 |
| NM_018059 | chr7.4922335.4924335 | 0.527881 | 0.044041 |
| NM_001322009 | chr7.6047659.6049659 | 1.580289 | 0.046944 |
| NM_001278559 | chr7.6675952.6677952 | 0.498276 | 0.045577 |
| NM_001281450 | chr7.72741085.72743085 | 0.538565 | 0.036749 |
| NM_001135211 | chr7.72741154.72743154 | 0.570207 | 0.04334 |
| NM_001306141 | chr7.75121946.75123946 | 0.66471 | 0.044041 |
| NM_000941 | chr7.75543419.75545419 | 1.984067 | 0.035608 |
| NM_001110354 | chr7.76053271.76055271 | 1.528203 | 0.042047 |
| NM_001164759 | chr7.766313.768313 | 0.404784 | 0.036749 |
| NM_020879 | chr7.76750933.76752933 | 1.604377 | 0.044041 |
| NM_021723 | chr7.87562457.87564457 | 1.612206 | 0.042047 |
| NM_001195543 | chr7.99194688.99196688 | 0.564907 | 0.038543 |
| NM_001185 | chr7.99572735.99574735 | 0.654316 | 0.034724 |
| NM_001135702 | chr8.101961799.101963799 | 0.385089 | 0.034724 |
| NM_003301 | chr8.110098652.110100652 | 1.54463 | 0.040468 |
| NM_001099677 | chr8.12868772.12870772 | 1.831898 | 0.044041 |
| NM_001323557 | chr8.132928000.132930000 | 1.537762 | 0.042396 |
| NM_177476 | chr8.143857438.143859438 | 1.564342 | 0.046944 |
| NM_001026213 | chr8.143960236.143962236 | 0.655597 | 0.04535 |
| NM_002347 | chr8.144240457.144242457 | 0.398804 | 0.040468 |
| NM_001135655 | chr8.144240755.144242755 | 0.407104 | 0.036749 |
| NM_001100878 | chr8.144653928.144655928 | 0.61271 | 0.045577 |
| NM_001280557 | chr8.145637975.145639975 | 0.576831 | 0.040468 |
| NM_147203 | chr8.17751913.17753913 | 1.984318 | 0.036645 |
| NM_004467 | chr8.17752047.17754047 | 2.054367 | 0.036645 |
| NM_014867 | chr8.1921043.1923043 | 0.4161 | 0.045577 |
| NM_018411 | chr8.21987565.21989565 | 0.406314 | 0.032921 |
| NM_018688 | chr8.22525661.22527661 | 0.452508 | 0.036749 |
| NM_001831 | chr8.27471328.27473328 | 0.646534 | 0.036645 |
| NM_015254 | chr8.29119610.29121610 | 0.449779 | 0.046944 |
| NM_001160004 | chr8.32404727.32406727 | 0.653283 | 0.045577 |
| NM_001164234 | chr8.38088008.38090008 | 0.466106 | 0.038643 |
| NM_001164232 | chr8.38088470.38090470 | 0.513951 | 0.046944 |
| NM_178819 | chr8.41434706.41436706 | 2.041965 | 0.038543 |
| NM_001321773 | chr8.50823232.50825232 | 1.53766 | 0.04535 |
| NM_001282904 | chr8.54163257.54165257 | 0.561227 | 0.034724 |
| NM_001286675 | chr8.54763367.54765367 | 1.555879 | 0.034724 |
| NM_173519 | chr8.62199524.62201524 | 0.615219 | 0.04334 |
| NM_001147 | chr8.6419784.6421784 | 0.587852 | 0.045577 |
| NM_001926 | chr8.6782598.6784598 | 1.63699 | 0.036269 |
| NM_001291339 | chr8.67995082.67997082 | 1.593238 | 0.04334 |
| NM_006421 | chr8.68254912.68256912 | 2.111125 | 0.04334 |
| NM_001349476 | chr8.69241607.69243607 | 1.914848 | 0.038643 |
| NM_001288574 | chr8.72273467.72275467 | 1.605754 | 0.034724 |
| NM_001286777 | chr8.75911388.75913388 | 1.852052 | 0.038543 |
| NM_024721 | chr8.77592514.77594514 | 1.550481 | 0.042047 |
| NM_194284 | chr8.8558665.8560665 | 2.118224 | 0.045577 |
| NM_004929 | chr8.91094109.91096109 | 1.690943 | 0.034724 |
| NM_203390 | chr8.94752224.94754224 | 0.579018 | 0.036749 |
| NM_017697 | chr8.95652363.95654363 | 0.439336 | 0.034724 |
| NM_018407 | chr8.98786808.98788808 | 1.735859 | 0.046944 |
| NM_001256312 | chr8.99953799.99955799 | 2.150898 | 0.034724 |
| NM_002839 | chr9.10611723.10613723 | 1.650517 | 0.046944 |
| NM_001004481 | chr9.107366908.107368908 | 1.573252 | 0.034724 |
| NM_001244713 | chr9.110046049.110048049 | 1.82151 | 0.036749 |
| NM_001080551 | chr9.114520813.114522813 | 1.772592 | 0.036749 |
| NM_001329590 | chr9.115479429.115481429 | 1.681755 | 0.045577 |
| NM_001859 | chr9.115982807.115984807 | 0.573962 | 0.036749 |
| NM_002160 | chr9.117879536.117881536 | 1.512065 | 0.034724 |
| NM_001166168 | chr9.127019529.127021529 | 1.586184 | 0.045577 |
| NM_203403 | chr9.12774011.12776011 | 1.60546 | 0.041292 |
| NM_005489 | chr9.130532648.130534648 | 1.845979 | 0.036749 |
| NM_016520 | chr9.132596572.132598572 | 1.812787 | 0.038643 |
| NM_001110303 | chr9.132596695.132598695 | 1.867851 | 0.036645 |
| NM_006059 | chr9.133883503.133885503 | 0.544596 | 0.035608 |
| NM_001316898 | chr9.135752706.135754706 | 1.738821 | 0.034724 |
| NM_001317959 | chr9.135753198.135755198 | 1.765823 | 0.034724 |
| NM_001316900 | chr9.135753277.135755277 | 1.604737 | 0.04334 |
| NM_001242370 | chr9.136324086.136326086 | 0.591983 | 0.042396 |
| NM_001282611 | chr9.137978513.137980513 | 0.639161 | 0.04535 |
| NM_001145639 | chr9.139246508.139248508 | 1.54705 | 0.045577 |
| NM_001282946 | chr9.139304024.139306024 | 0.552353 | 0.04535 |
| NM_152421 | chr9.139606023.139608023 | 0.637698 | 0.04334 |
| NM_207309 | chr9.139970952.139972952 | 1.941876 | 0.038643 |
| NM_006088 | chr9.140134710.140136710 | 2.028185 | 0.041292 |
| NM_152286 | chr9.140444021.140446021 | 1.544468 | 0.039622 |
| NM_001354125 | chr9.14692544.14694544 | 0.646209 | 0.04334 |
| NM_001271829 | chr9.16252105.16254105 | 1.70697 | 0.041292 |
| NM_024013 | chr9.21439452.21441452 | 1.796812 | 0.042047 |
| NM_001351474 | chr9.23821553.23823553 | 0.593253 | 0.044041 |
| NM_001351469 | chr9.23822130.23824130 | 0.60045 | 0.041292 |
| NM_001271706 | chr9.24544674.24546674 | 1.535368 | 0.044041 |
| NM_024761 | chr9.27528850.27530850 | 0.619927 | 0.044041 |
| NM_001195622 | chr9.32551626.32553626 | 2.280111 | 0.041292 |
| NM_002504 | chr9.33289414.33291414 | 0.459364 | 0.040468 |
| NM_001128227 | chr9.36276053.36278053 | 0.628176 | 0.04535 |
| NM_012203 | chr9.37421706.37423706 | 1.543968 | 0.04535 |
| NM_016042 | chr9.37784089.37786089 | 0.61574 | 0.041292 |
| NM_004170 | chr9.4489426.4491426 | 0.619356 | 0.04334 |
| NM_025239 | chr9.5509544.5511544 | 0.541369 | 0.036269 |
| NM_005511 | chr9.5889908.5891908 | 0.608789 | 0.040468 |
| NM_001146696 | chr9.6719862.6721862 | 1.683932 | 0.035608 |
| NM_138691 | chr9.75135716.75137716 | 0.666194 | 0.04334 |
| NM_001282688 | chr9.91932387.91934387 | 1.870955 | 0.042396 |
| NM_001282690 | chr9.91932765.91934765 | 1.571317 | 0.044041 |
| NM_001393 | chr9.95297374.95299374 | 2.764312 | 0.034724 |

FC=fold change. FDR=false positive rate. ALN=axillary lymph node.

**Table S2. The genes in PPCNM**

| Number | RefID | Gene symbol | PMID |
| --- | --- | --- | --- |
| 1 | NM_001289990 | ATP6V0E2 | / |
| 2 | NM_003670 | BHLHE40 | 30285805; 29704436 |
| 3 | NM_018688 | BIN3 | / |
| 4 | NM_152587 | C11orf65 | / |
| 5 | NM_016520 | C9orf78 | / |
| 6 | NM_001033677 | CABP1 | / |
| 7 | NM_001201372 | CCDC136 | 15112360 |
| 8 | NM_001159588 | CD109 | 32133706; 32007357; 33565282 |
| 9 | NM_001320638 | CD53 | 26631805; 10733097 |
| 10 | NM_001321927 | CKMT1A | 29753758 |
| 11 | NM_004645 | COIL | / |
| 12 | NM_001198853 | CYP2C8 | 25406731 |
| 13 | NM_001318835 | DHRS2 | 29106393 |
| 14 | NM_133637 | DQX1 | / |
| 15 | NM_016337 | EVL | 18357390; |
| 16 | NM_173526 | FAM71D | / |
| 17 | NM_001354956 | FGF1 | 30429477; 25124967; 18041768 |
| 18 | NM_004467 | FGL1 | 29845203 |
| 19 | NM_198897 | FIBP | / |
| 20 | NM_006653 | FRS3 | / |
| 21 | NM_001145667 | GLG1 | 30988423 |
| 22 | NM_001123375 | HIST2H3D | 29655996 |
| 23 | NM_002126 | HLF | 27522003; 32289442 |
| 24 | NM_005546 | ITK | 32025027 |
| 25 | NM_016195 | KIF20B | 29573464 |
| 26 | NM_001322351 | LMAN2L | / |
| 27 | NM_001349205 | LPIN1 | 33203880 |
| 28 | NM_001410 | MEGF8 | / |
| 29 | NM_030811 | MRPS26 | / |
| 30 | NM_170721 | MSI2 | 32606289 |
| 31 | NM_002489 | NDUFA4 | 33117603 |
| 32 | NM_001077494 | NFKB2 | 12160329;31346317 |
| 33 | NM_018297 | NGLY1 | / |
| 34 | NM_002516 | NOVA2 | 3336482; 31832068 |
| 35 | NM_001005324 | OR10V1 | / |
| 36 | NM_000288 | PEX7 | / |
| 37 | NM_001201545 | RBM15 | 31142332 |
| 38 | NM_001168398 | SLC35A1 | / |
| 39 | NM_001348905 | SLC35A5 | / |
| 40 | NM_001143824 | SLC38A4 | / |
| 41 | NM_001311178 | SLMAP | / |
| 42 | NM_001350738 | SRPK2 | 29587239 |
| 43 | NM_001359094 | TDRG1 | 31742752; 31164797 |
| 44 | NM_014777 | URB2 | / |
| 45 | NM_001110303 | USP20 | 32943575 |
| 46 | NM_019086 | VSIG10 | / |
| 47 | NM_001348170 | WBP2 | 33837178 |
| 48 | NM_001331097 | ZNF114 | / |

PMID showed the metastasis-related literatures of this gene.

**Table S3. The predictive efficacy of PPCNM in different tumor fraction**

| Tumor Fraction | AUC (95% CI) | Acc | Sen | Spe | *P* (g1 vs other g) | *P* (g2 vs other g) | *P* (g1 vs other g) |
| --- | --- | --- | --- | --- | --- | --- | --- |
| <5%  (group1) | 0.921(0.859-0.983) | 92.2 | 91.2 | 93.0 | / | / | / |
| 5-10%  (group2) | 0.910(0.851-0.968) | 91.2 | 97.9 | 84.1 | 0.797 | / | / |
| 10-15%  (group3) | 0.870(0.815-0.925) | 87.0 | 87.3 | 86.7 | 0.228 | 0.331 | / |
| >15%  (group4) | 0.950(0.852-1) | 93.8 | 90.0 | 100.0 | 0.628 | 0.496 | 0.175 |

Acc=accuracy. Sen=sensitivity. Spe=specificity. Other g=other groups. The *P*-value of the AUC comparison between different groups was calculated using the function of pROC package.

**Table S4. Predictive efficacy of the PPCNM with characteristics**

| Combinations | Training cohort | | |  | Validation cohort | | |  | All cohort | | |
| --- | --- | --- | --- | --- | --- | --- | --- | --- | --- | --- | --- |
|  | ACC | SEN | SPE |  | ACC | SEN | SPE |  | ACC | SEN | SPE |
| +ER | 0.909 | 0.920 | 0.897 |  | 0.810 | 0.796 | 0.824 |  | 0.879 | 0.883 | 0.875 |
| +PR | 0.909 | 0.929 | 0.889 |  | 0.820 | 0.837 | 0.804 |  | 0.882 | 0.901 | 0.863 |
| +Her2 | 0.904 | 0.912 | 0.897 |  | 0.820 | 0.837 | 0.804 |  | 0.879 | 0.889 | 0.869 |
| +Ki67 | 0.883 | 0.885 | 0.880 |  | 0.810 | 0.796 | 0.824 |  | 0.861 | 0.858 | 0.863 |
| +ER/PR | 0.887 | 0.885 | 0.889 |  | 0.820 | 0.837 | 0.804 |  | 0.867 | 0.870 | 0.863 |
| +ER/Her2 | 0.870 | 0.867 | 0.872 |  | 0.800 | 0.816 | 0.784 |  | 0.848 | 0.852 | 0.845 |
| +ER/Ki67 | 0.861 | 0.867 | 0.855 |  | 0.800 | 0.760 | 0.804 |  | 0.842 | 0.846 | 0.839 |
| +PR/Her2 | 0.878 | 0.885 | 0.872 |  | 0.810 | 0.836 | 0.784 |  | 0.858 | 0.870 | 0.845 |
| +PR/Ki67 | 0.883 | 0.894 | 0.872 |  | 0.810 | 0.816 | 0.804 |  | 0.861 | 0.870 | 0.851 |
| +Her2/Ki67 | 0.861 | 0.867 | 0.854 |  | 0.810 | 0.776 | 0.843 |  | 0.845 | 0.840 | 0.851 |
| +ER/PR/Her2 | 0.865 | 0.849 | 0.880 |  | 0.810 | 0.816 | 0.804 |  | 0.848 | 0.840 | 0.851 |
| +ER/PR/Ki67 | 0.857 | 0.850 | 0.863 |  | 0.820 | 0.816 | 0.824 |  | 0.845 | 0.840 | 0.851 |
| +ER/Her2/Ki67 | 0.856 | 0.850 | 0.863 |  | 0.820 | 0.816 | 0.824 |  | 0.845 | 0.840 | 0.851 |
| +ER/PR/Her2/Ki67 | 0.848 | 0.850 | 0.846 |  | 0.810 | 0.776 | 0.843 |  | 0.836 | 0.827 | 0.845 |

ACC=accuracy. SEN=sensitivity. SPE=specificity. ER=estrogen receptor. PR=progesterone receptor. Her2=human epidermal growth factor receptor 2.
